# Endothelial force sensing signals to parenchymal cells to regulate bile and plasma lipids

**DOI:** 10.1126/sciadv.adq3075

**Published:** 2024-09-27

**Authors:** Laeticia Lichtenstein, Chew W. Cheng, Muath Bajarwan, Elizabeth L. Evans, Hannah J. Gaunt, Fiona Bartoli, Eulashini Chuntharpursat-Bon, Shaili Patel, Charalampos Konstantinou, T. Simon Futers, Melanie Reay, Gregory Parsonage, J. Bernadette Moore, Justine Bertrand-Michel, Piruthivi Sukumar, Lee D. Roberts, David J. Beech

**Affiliations:** ^1^ School of Medicine, University of Leeds, Leeds LS2 9JT, UK.; ^2^ School of Food Science and Nutrition, University of Leeds, Leeds LS2 9JT, UK.; ^3^ Department of Hepatobiliary and Transplant Surgery, St James's University Hospital, Leeds LS9 7TF, UK.; ^4^ MetaToul-Lipidomics Facility, INSERM UMR1048, Toulouse, France.; ^5^ Institut des Maladies Métaboliques et Cardiovasculaires, UMR 1297/I2MC, INSERM, Toulouse, France.

## Abstract

How cardiovascular activity interacts with lipid homeostasis is incompletely understood. We postulated a role for blood flow acting at endothelium in lipid regulatory organs. Transcriptome analysis was performed on livers from mice engineered for deletion of the flow-sensing PIEZO1 channel in endothelium. This revealed unique up-regulation of *Cyp7a1*, which encodes the rate-limiting enzyme for bile synthesis from cholesterol in hepatocytes. Consistent with this effect were increased gallbladder and plasma bile acids and lowered hepatic and plasma cholesterol. Elevated portal fluid flow acting via endothelial PIEZO1 and genetically enhanced PIEZO1 conversely suppressed *Cyp7a1*. Activation of hepatic endothelial PIEZO1 channels promoted phosphorylation of nitric oxide synthase 3, and portal flow-mediated suppression of *Cyp7a1* depended on nitric oxide synthesis, suggesting endothelium-to-hepatocyte coupling via nitric oxide. *PIEZO1* variants in people were associated with hepatobiliary disease and dyslipidemia. The data suggest an endothelial force sensing mechanism that controls lipid regulation in parenchymal cells to modulate whole-body lipid homeostasis.

## Teaser

Vascular blood flow sensor regulates lipid homeostasis and body fat.

## INTRODUCTION

Cardiovascular disease is the leading cause of death worldwide (*1*). A major step forward in the fight against it was the recognition of importance of hyperlipidemia, which led to successful strategies such as statin medication to lower cholesterol (*2*, *3*). The disease remains unsolved, however, and presents an ever-increasing global challenge (*4*). Ongoing research focuses on an improved generation of therapies aimed at lowering triglycerides as well as cholesterol to protect against cardiovascular disease and its comorbidities such as nonalcoholic fatty liver disease (*4*, *5*). Identification of previously unrecognized mechanisms controlling such lipids could offer advanced opportunities for understanding the disease and developing therapies.

A major factor increasing the risk of cardiovascular disease is sedentary lifestyle. Interruption of sedentary behavior by physical activity attenuates postprandial lipid elevation (*6*), which suggests endogenous mechanically regulated mechanisms that suppress hyperlipidemia. A key mechanical factor could be shear stress at the endothelium, which is generated by blood flow and modulated when blood flow redistributes in physical activity (*7*, *8*). A potential molecular mediator is the PIEZO1 protein, which is an exceptional eukaryotic mechanical force sensor (*9*–*12*) that is expressed in endothelium and is pivotal in the sensing of shear stress (*13*–*25*).

Three PIEZO1s associate to form an ion channel of 114 membrane-spanning segments that, in its closed state, indents the membrane and awaits force with its lipid-embedded blades (*11*). The channel is thus primed for almost instantaneous coupling of force into transmembrane ion flux, thereby regulating cell function (*11*, *26*–*29*). Endothelial PIEZO1 is already recognized for its involvement in vascular development and structure (*15*), exercise-dependent blood pressure regulation (*17*), atherosclerosis (*19*), angiogenesis (*15*, *30*), vascular permeability (*31*, *32*), lymphatic valve formation (*33*), leukocyte diapedesis (*34*), muscle capillary density (*35*), and physical activity performance (*35*). Moreover, it is a powerful positive modulator of one of the most important cardiovascular and metabolic mediators, nitric oxide (*15*, *20*, *25*, *36*).

PIEZO1 is functional in endothelial cells of the liver (*14*, *15*, *17*, *37*, *38*), the primary organ for lipid regulation and cholesterol synthesis (*39*). We therefore tested whether endothelial PIEZO1 affects hepatic and thus systemic lipid homeostasis, beginning with unbiased analysis of gene expression in the liver.

## RESULTS

### Deletion of endothelial PIEZO1 uniquely up-regulates *Cyp7a1* in the liver

**Fig. 1.**
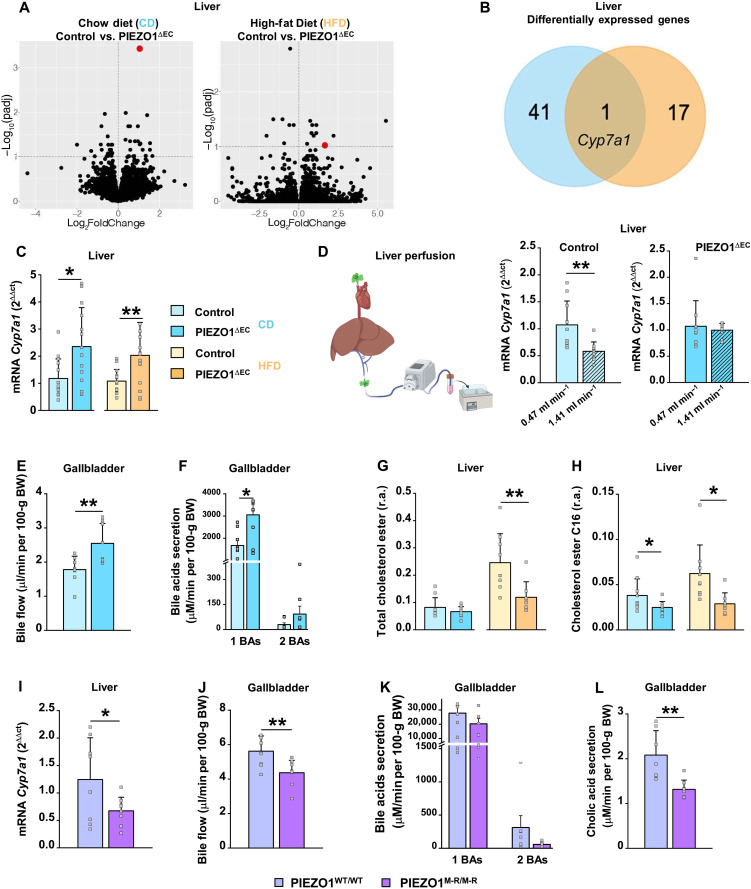
Regulation of *Cyp7a1* and hepatobiliary lipid homeostasis. (**A** to **H**) Data for 20-week-old mice fed CD or HFD for 8 weeks, comparing control and PIEZO1^∆EC^ mice. (**I** to **L**) Data for 8-week-old mice fed CD, comparing wild-type PIEZO1^WT/WT^ and PIEZO1^M-R/M-R^ mice. (A) Volcano plots for differentially expressed genes (DEGs), with adjusted *P* value (padj) <0.1 (*n* = 5). Red dot, *Cyp7a1*. (B) Venn diagram for DEGs in CD and HFD. (C) mRNA abundance determined by qPCR for *Cyp7a1* in liver of control and PIEZO1^∆EC^ mice fed CD (*n* = 14) or HFD (*n* = 15), normalized to *rps19* mRNA abundance. (D) Left: Experimental arrangement. Right: Liver *Cyp7a1* qPCR data from liver perfusion in control mice (no hatch, 0.47 ml min^−1^; hatched, 1.41 ml min^−1^, *n* = 10) and PIEZO1^∆EC^ mice at 0.47 ml min^−1^ (*n* = 10) and at 1.41 ml min^−1^ (*n* = 9). (E and F) Gallbladder cannulation data for control and PIEZO1^∆EC^ mice on CD, showing bile flow (E) (*n* = 8) and primary bile acids (1 BAs) and secondary bile acids (2 BAs) secretion (F) (*n* = 8). (G) Relative abundance (r.a.) of total cholesterol ester in liver (CD *n* = 10 each; HFD *n* = 9 control and *n* = 8 PIEZO1^∆EC^ mice). (H) As for (G) but cholesterol ester C16 (CD *n* = 10 mice; HFD *n* = 9 mice). (I to L) For PIEZO1^WT/WT^ (*n* = 8) and PIEZO1^M-R/M-R^ (*n* = 8 to 9) measurements of (I) hepatic *Cyp7a1* mRNA abundance normalized to *rps19* mRNA, (J) bile flow, (K) 1 BAs and 2 BAs secretion, and (L) cholic acid secretion. Summary data are means ± SD with superimposed individual data points. Unpaired *t* test. Statistically significant differences: *P < 0.05; **P < 0.01. (D) Created with BioRender.com.

Mice genetically engineered for conditional endothelial-specific deletion of PIEZO1 at the adult stage (*14*, *17*) (fig. S1) were studied. Because lipid homeostasis depends on dietary fat, matched control and endothelial PIEZO1–deleted mice (PIEZO1^∆EC^ mice) were compared in conditions of low-fat chow diet (CD) and high-fat (60%) diet (HFD). RNA sequencing was used to compare liver transcriptomes. Increased *Cyp7a1* expression was the only change that was independent of dietary fat (Fig. 1, A and B). The increase was validated by quantitative polymerase chain reaction (qPCR) in independent mice (Fig. 1C). *Cyp7a1* encodes Cytochrome P450 7A1 (CP7A1), which catalyzes cholesterol catabolism and classical bile acid synthesis (*40*), accounting for a large fraction of bile production (*41*). Transcriptional regulation is a key aspect of its control (*42*). Consistent with its exclusive localization to hepatocytes (*40*), there was no expression in liver endothelial cells (fig. S2). Other genes involved in bile acid synthesis (*Akr1k1*, *Cyp8b1*, *Cyp2c70*, and *Cyp27a1*), export (*Abcb11* and *Abcc2*), reabsorption (*Abcc3* and *Slco1a1*), conjugation (*Scl27a5* and *Baat*), and signaling (*Nr1h4*, *Nr0b2*, and *Fgfr4*) were unaffected (table S1). Although altered expression of *Slc10a1* (a gene encoding a sodium/bile acid cotransporter) was found, this was only in the HFD condition (table S1). The data suggest a connection between mechanical force acting at the endothelium and regulation of *Cyp7a1*.

### Portal flow down-regulates *Cyp7a1*

To test whether mechanical force acting at hepatic endothelium regulates *Cyp7a1*, we cannulated mice to artificially control fluid flow in the portal vein, which mediates about 75% of the liver’s blood flow (*43*). Portal flow in mice is estimated at 1.6 to 2.3 ml min^−1^ (*44*), decreasing with whole-body physical activity and increasing after a meal (*45*, *46*). Comparison of 0.47 and 1.41 ml min^−1^ flow rates revealed less *Cyp7a1* expression at the higher flow rate (1.41 ml min^−1^) and absence of this effect in PIEZO1^∆EC^ mice (Fig. 1D). The data suggest that fluid flow acting via endothelial PIEZO1 inhibits *Cyp7a1*.

### Deletion of endothelial PIEZO1 up-regulates bile production

To test for functional consequences of the *Cyp7a1* regulation, we performed gallbladder cannulation to access bile. Bile flow was increased in PIEZO1^∆EC^ mice (Fig. 1E), consistent with the up-regulated *Cyp7a1* expression. Total secreted primary bile acids were increased (Fig. 1F), as were individual primary bile acids (e.g., cholate and taurocholate; table S2). Total secondary bile acids were unchanged (Fig. 1F), but individual secondary bile acids (e.g., taurodeoxycholate and ω-tauromuricholate; table S2) were increased. The data suggest functional consequences of PIEZO1-related *Cyp7a1* up-regulation for bile composition and flow.

### Deletion of endothelial PIEZO1 down-regulates hepatic cholesterol

CP7A1 catalyzes bile acid synthesis from cholesterol (*41*, *42*), so less cholesterol is expected in the liver when there is more bile synthesis. Consistent with this expectation, livers of PIEZO1^∆EC^ mice contained less total cholesterol ester, although only in the HFD condition (Fig. 1G). Cholesteryl palmitate was, however, less in both dietary conditions (Fig. 1H), indicating specific regulation of this subspecies. The data suggest significance of endothelial PIEZO1 for cholesterol abundance, consistent with its effect on *Cyp7a1*.

### Genetic enhancement of PIEZO1 down-regulates *Cyp7a1*

To test the role of PIEZO1 independently of PIEZO1^∆EC^ mice, we used mice carrying a mutation that increases PIEZO1 channel activity (PIEZO1^M-R/M-R^ mice) (*47*). These mice were studied in the CD condition only. In these mice, *Cyp7a1* expression (Fig. 1I) and bile flow (Fig. 1J) were less. Total primary and secondary bile acids were unchanged (Fig. 1K), but cholate and five other bile acids were less (Fig. 1L and table S3). The data support the idea that PIEZO1 is a negative regulator of *Cyp7a1* and bile synthesis.

### Coupling to *Cyp7a1* depends on nitric oxide synthesis

**Fig. 2.**
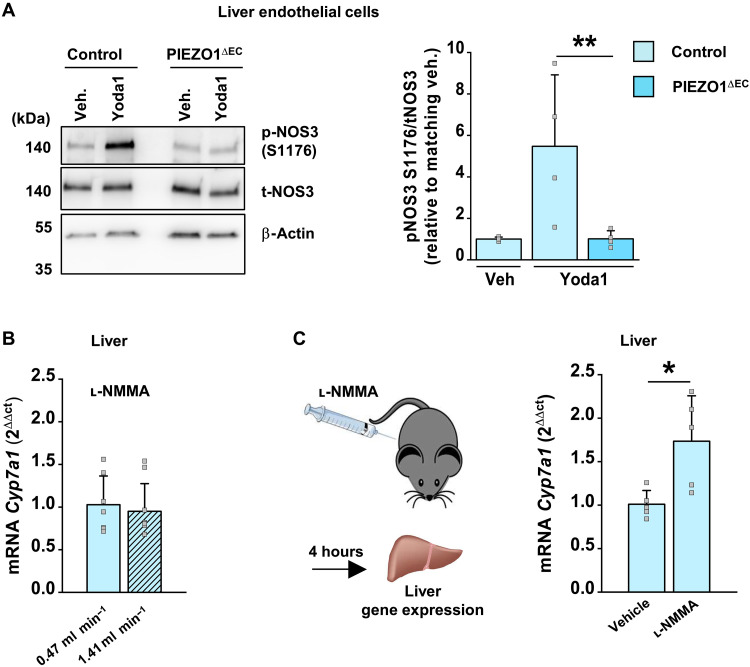
Nitric oxide synthesis is required for coupling to *Cyp7a1*. (**A**) Left: Example Western blot for liver microvascular endothelial cell proteins from control and PIEZO1^∆EC^ mice after 1-min treatment with 2 μM Yoda1 or the vehicle (veh., dimethyl sulfoxide) control. Blots were probed for NOS3 phosphorylation at S1176 (p-NOS3), total NOS3 (t-NOS3) and the loading control protein, β-actin. Right: Quantification for experiments of the type exemplified, expressed as phospho-NOS3 intensity relative to total NOS3 intensity and normalized to matching vehicle condition (*n* = 4 each group). (**B**) Bar chart showing liver *Cyp7a1* qPCR data from liver perfusion experiments in control mice of the type described in Fig. 1D comparing the effects of the two perfusion rates, both with 0.3 mM l-NMMA in the perfusate (0.47 ml min^−1^, *n* = 7 mice; hatched, 1.41 ml min^−1^, *n* = 9 mice). (**C**) Left: Schematic of injection of l-NMMA and harvesting of the liver for later mRNA quantification. Right: Bar chart showing liver *Cyp7a1* qPCR data for wild-type mice fed CD and injected retro-orbitally with 72 μl of 1 mM l-NMMA per 100 g of body weight or vehicle control [phosphate-buffered saline (PBS)] (*n* = 5 each group). Summary data are means ± SD and superimposed individual data points, some of which are overlapping. Unpaired *t* test. Statistically significant differences: *P < 0.05; **P < 0.01.

We considered how PIEZO1 in endothelium might signal to *Cyp7a1* in hepatocytes. In nonhepatic endothelial cells, PIEZO1 promotes phosphorylation of nitric oxide synthase 3 (NOS3), increasing nitric oxide production (*15*, *20*, *25*). In hepatocytes, nitric oxide inhibits expression of *Cyp7a1* via a nitrosylation pathway (*48*, *49*). Nitric oxide is readily diffusible (*36*) and so could link endothelium to hepatocyte genes. We first quantified phosphorylation of NOS3 in total liver lysate but found no differences between control and PIEZO1^∆EC^ mice (fig. S3). However, NOS3 is strongly expressed in hepatocytes (*50*, *51*), the dominant cells of the liver that greatly outnumber endothelial cells. Therefore, we isolated hepatic endothelial cells to specifically investigate endothelial NOS3 and used the PIEZO1 agonist Yoda1 to specifically activate PIEZO1 independently of other potential force sensors (*17*). Strong PIEZO1-dependent NOS3 phosphorylation was now detected in response to Yoda1 (Fig. 2A), suggesting endothelial-specific regulation of NOS3. To explore relevance of the signaling when there is mechanical stimulus for the channels in the hepatic circulation, a substrate inhibitor of nitric oxide synthesis [N^G^-methyl-l-arginine (l-NMMA)] was added to the portal perfusate. High flow-induced inhibition of *Cyp7a1* was now absent (Fig. 2B), consistent with nitric oxide synthesis being required for the flow-induced down-regulation of *Cyp7a1*. Heme-coordinated *Nr1d1* (*52*) was similarly regulated (fig. S4), consistent with nitric oxide increasing with flow. To determine the relevance in freely moving mice, we administered l-NMMA systemically. There was increased *Cyp7a1* expression (Fig. 2C) without effect on the expression of other genes tested (table S4). The data suggest that nitric oxide synthesis is critical in the link between flow-activated PIEZO1 in endothelium and *Cyp7a1* in hepatocytes.

### Plasma lipid effects match and diverge from hepatic effects

**Fig. 3.**
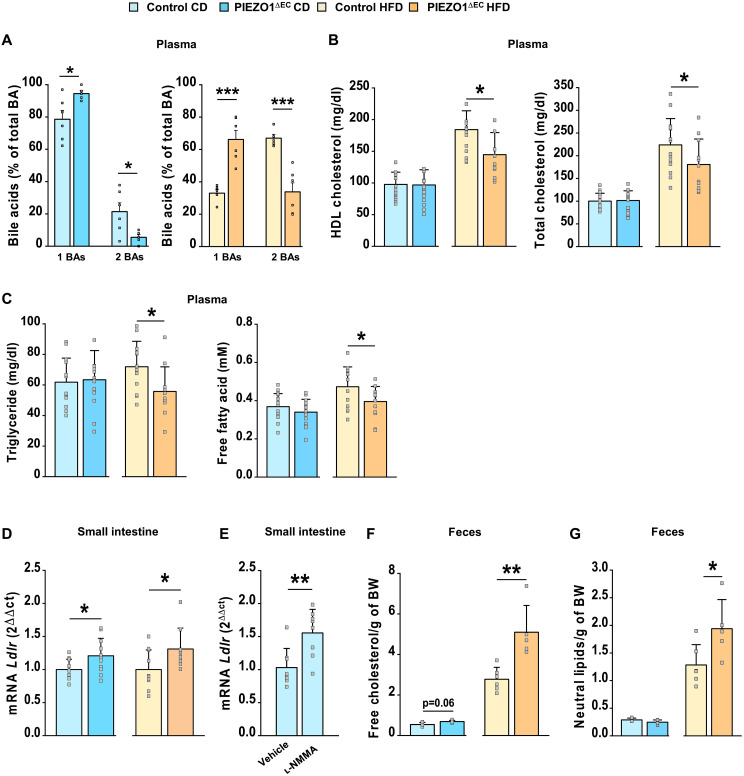
Whole-body lipid effects beyond regulation of *Cyp7a1*. Data for 20-week-old mice fed CD (blue) or HFD (orange) for 8 weeks (lighter color, control mice; darker color, PIEZO1^∆EC^ mice. (**A**) Plasma primary and secondary bile acids (1 BAs and 2 BAs) of CD-fed mice on the left and HFD-fed mice on the right (*n* = 6 per group). (**B**) Plasma concentrations of HDL cholesterol (CD *n* = 17 control and *n* = 15 PIEZO1^∆EC^ mice; HFD *n* = 13 control and *n* = 11 PIEZO1^∆EC^ mice) and total cholesterol (CD *n* = 17 control and *n* = 16 PIEZO1^∆EC^ mice; HFD *n* = 14 control and *n* = 11 PIEZO1^∆EC^ mice). (**C**) Plasma triglycerides (CD *n* = 14 control and *n* = 15 PIEZO1^∆EC^ mice; HFD *n* = 12 control and *n* = 11 PIEZO1^∆EC^ mice) and free fatty acids (CD *n* = 17 control and *n* = 16 PIEZO1^∆EC^ mice; HFD *n* = 15 control and *n* = 14 PIEZO1^∆EC^ mice). (**D**) *Ldlr* mRNA abundance in distal small intestine (CD *n* = 10 control and *n* = 11 PIEZO1^∆EC^ mice; HFD *n* = 10 control and *n* = 9 PIEZO1^∆EC^ mice). (**E**) *Ldlr* mRNA abundance in distal small intestine of wild-type mice fed CD and injected retro-orbitally with 72 μl of 1 mM l-NMMA per 100 g of body weight or vehicle control (PBS) (*n* = 8). (**F**) Fecal free cholesterol per gram of body weight (*n* = 6 control and *n* = 5 PIEZO1^∆EC^ mice). (**G**) Fecal total neutral lipid per gram of body weight (*n* = 6 control and *n* = 5 PIEZO1^∆EC^ mice). Summary data are means ± SD with superimposed individual data points, some of which are overlapping. Unpaired *t* test. Statistically significant differences: *P < 0.05; **P < 0.01; ***P < 0.001.

Bile homeostasis is expected to affect whole-body lipid homeostasis (*42*, *53*), so we investigated beyond the hepatobiliary system to plasma. Total primary bile acids and individual bile acids were increased in the plasma of PIEZO1^∆EC^ mice (Fig. 3A and table S5), similar to the effects in the gallbladder (Fig. 1F). Total secondary bile acids were, however, decreased (Fig. 3A). High-density lipoprotein (HDL) cholesterol and total cholesterol were decreased in the HFD condition (Fig. 3B), partly aligning to the hepatic effect (Fig. 1, G and H). Effects of bile are complex (*54*), but bile emulsifies lipids in the intestine, thus potentially improving their availability for uptake to the circulation. However, in PIEZO1^∆EC^ mice (that have increased bile), plasma triglycerides and free fatty acids were unchanged in the CD condition and decreased in the HFD condition (Fig. 3C). The data suggest effects on plasma lipids that do not follow the hepatic phenotype.

### Deletion of endothelial PIEZO1 increases intestinal *Ldlr* expression and lipid excretion

The small intestine is another major organ of lipid regulation, closely integrating with the hepatobiliary system. To address its role, we quantified expression of selected genes known to regulate intestinal lipid homeostasis. Expression of only one of these genes, *Ldlr*, increased in distal small intestine of PIEZO1^∆EC^ mice in both diet conditions (Fig. 3D and table S6). Its expression was also increased by l-NMMA (Fig. 3E), similar to *Cyp7a1* in the liver. *Ldlr* encodes low-density lipoprotein receptor (LDLR), which is expressed on the basolateral membrane of the mucosal epithelium, where it mediates the uptake of circulating low-density lipoprotein (LDL). Consistent with this expression, there is increased fecal fat and cholesterol in mice that have increased expression of intestinal *Ldlr* (*55*). Therefore, we investigated lipids in feces of PIEZO1^∆EC^ mice, anticipating increases in their abundance. Fecal cholesterol and neutral lipids were indeed increased, although only in the HFD condition (Fig. 3, F and G). The data suggest that endothelial PIEZO1 regulates small intestine *Ldlr* expression and lipid excretion and therefore plasma lipids.

### Endothelial PIEZO1–deleted mice show reduced hepatic and visceral fat

**Fig. 4.**
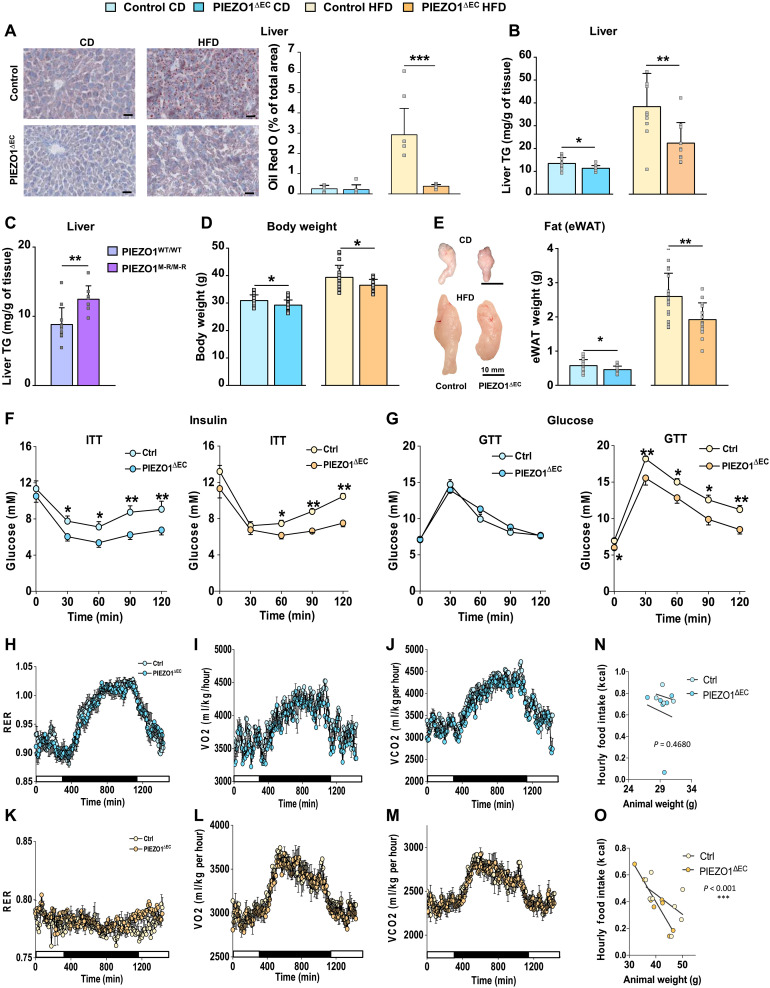
Impact on fat storage and insulin sensitivity. Data for 20-week-old mice fed CD (blue) or HFD (orange) for 8 weeks. (**A**) Left: Representative images of Oil Red O–stained liver sections of control and PIEZO1^∆EC^ mice. Scale bars, 3 mm. Right: Quantification of Oil Red O staining: *n* = 5 control and *n* = 7 PIEZO1^∆EC^ mice. (**B**) Hepatic triglyceride (TG) content in mice fed CD (*n* = 15 control and *n* = 13 PIEZO1^∆EC^) or HFD (*n* = 10 each). (**C**) Hepatic TG content in 8-week-old mice fed CD (*n* = 10 PIEZO1^WT/WT^, *n* = 7 PIEZO1^M-R/M-R^). (**D**) Body weights (*n* = 23) (**E**) Left: Representative images of epididymal white adipose tissue (eWAT). Scale bars, 10 mm. Right: Weights for eWAT (CD *n* = 18 control and *n* = 15 PIEZO1^∆EC^ mice; HFD *n* = 19 control and *n* = 14 PIEZO1^∆EC^ mice). (**F** and **G**) Plasma glucose after intraperitoneal insulin tolerance test (ITT) (F) or intraperitoneal glucose tolerance test (GTT) (G) for mice on CD (left) and HFD (right). (ITT: CD *n* = 15 control, *n* = 18 PIEZO1^∆EC^; HFD *n* = 13 control, *n* = 10 PIEZO1^∆EC^; GTT: CD *n* = 20 control, *n* = 21 PIEZO1^∆EC^; HFD *n* = 20 control, *n* = 18 PIEZO1^∆EC^). (**H** to **O**) Data for mice in CLAMS for four consecutive days; (H and K) for 20-week-old mice on CD (blue) or HFD (orange), showing (*n* = 4 to 8): RER; oxygen consumption (VO_2_; I and L) and carbon dioxide production (VCO_2_; J and M). (N and O) Food intake versus body weight of mice fed CD (N) and HFD (O). Summary data are means ± SD with superimposed individual data points, some of which are overlapping. Unpaired *t* test. Statistically significant differences: *P < 0.05; **P < 0.01; ***P < 0.001.

Increases in lipid excretion and reduced plasma lipids might reduce stored fat. Consistent with this expectation, PIEZO1^∆EC^ mice were protected against HFD-associated hepatic fat deposits (Fig. 4A). Hepatic triglycerides were reduced under the CD and HFD conditions (Fig. 4B). Conversely, in PIEZO1 gain-of-function (PIEZO1^M-R/M-R^) mice, hepatic triglycerides were increased (Fig. 4C). In both diet conditions, PIEZO1^∆EC^ mice weighed less (Fig. 4D) and had less epididymal white adipose tissue (Fig. 4E). Because adiposity associates with diabetes, we performed insulin and glucose tolerance tests. Insulin more effectively lowered plasma glucose in PIEZO1^∆EC^ mice, suggesting improved insulin sensitivity (Fig. 4F), which may be related to the reduced adiposity. Glucose control was improved in the HFD condition (Fig. 4G). Weight loss can arise from increased whole-body energetics, but respiratory exchange ratio (RER), VO_2_, VCO_2_, and heat generation were unchanged (Fig. 4, H to M). Food intake was less only in the most obese mice (Fig. 4, N and O, and fig. S5). Fecal bacteria were affected by HFD but not PIEZO1 deletion (fig. S6). The data suggest effects of endothelial PIEZO1 on fat storage and insulin sensitivity.

### Exploration of PIEZO1-associated mutations suggests relevance to humans

Mechanisms in mice do not always correspond to humans, but fluid flow responses of endothelial cells from human liver specimens are PIEZO1 dependent (*15*), suggesting PIEZO1 functionality in hepatic endothelial cells of people. To further investigate the human relevance, we interrogated genome population data using a *PIEZO1*-focused candidate approach. UK Biobank data, which are primarily from white British people, indicated associations of *PIEZO1* variants with death attributed to fatty liver, alcoholic liver disease, and liver biliary pancreas problems (Table 1). National Human Genome Research Institute–European Bioinformatics Institute (NHGRI-EBI) data from more diverse populations associated variants with body mass index–adjusted waist circumference (Table 2). Other datasets (i.e., FinnGen and the Cardiovascular Disease Knowledge Portal) associated variants with toxic liver disease, nonalcoholic fatty liver disease, LDL cholesterol and total cholesterol (table S7). The data suggest PIEZO1 relevance to hepatobiliary function and lipid homeostasis in humans.

**Table 1.** *PIEZO1 *variants associate with hepatobiliary parameters. Data are from UK Biobank. rsID, reference single nucleotide polymorphism (SNP) cluster identification. Five SNPs are missense variants affecting PIEZO1 protein sequence: R2390Q (rs377011269); V250I (rs115486714); A1497V (rs942117829); N1332T (rs201091400); and F1247C (rs907173644).

rsID	*P* value	Phenotype	Database	Variant type
rs565024125	1.26 × 10^−14^	Cause of death: fatty liver	UK Biobank	Intronic
rs377011269	2.21 × 10^−05^	Alcoholic liver diseases	UK Biobank	Missense
rs759885882	4.29 × 10^−07^	Alcoholic liver diseases	UK Biobank	Intronic
rs115486714	6.85 × 10^−08^	Alcoholic liver diseases	UK Biobank	Missense
rs915624083	3.18 × 10^−05^	Liver biliary pancreas problem	UK Biobank	Intronic
rs1044492299	1.65 × 10^−06^	Liver biliary pancreas problem	UK Biobank	Intronic
rs95793364	9.78 × 10^−06^	Liver biliary pancreas problem	UK Biobank	Intronic
rs747295957	3.18 × 10^−05^	Liver biliary pancreas problem	UK Biobank	Intronic
rs942117829	6.35 × 10^−06^	Liver biliary pancreas problem	UK Biobank	Missense
rs201091400	4.74 × 10^−06^	Liver biliary pancreas problem	UK Biobank	Missense
rs1908139729	9.57 × 10^−06^	Liver biliary pancreas problem	UK Biobank	Intronic
rs907173644	2.85 × 10^−05^	Liver biliary pancreas problem	UK Biobank	Missense

**Table 2.** *PIEZO1* variants associate with body weight parameters. Data are from NHGRI-EBI. rsID, reference single nucleotide polymorphism (SNP) cluster identification.

rsID	*P* value	Phenotype	Database	Variant type
rs4782429	1 × 10^−11^	BMI-adjusted waist circumference	NHGRI-EBI	Splice
rs7192637	8 × 10^−11^	BMI-adjusted waist circumference	NHGRI-EBI	Intronic
rs9788834	3 × 10^−8^	BMI-adjusted waist circumference	NHGRI-EBI	Intronic

## DISCUSSION

**Fig. 5.**
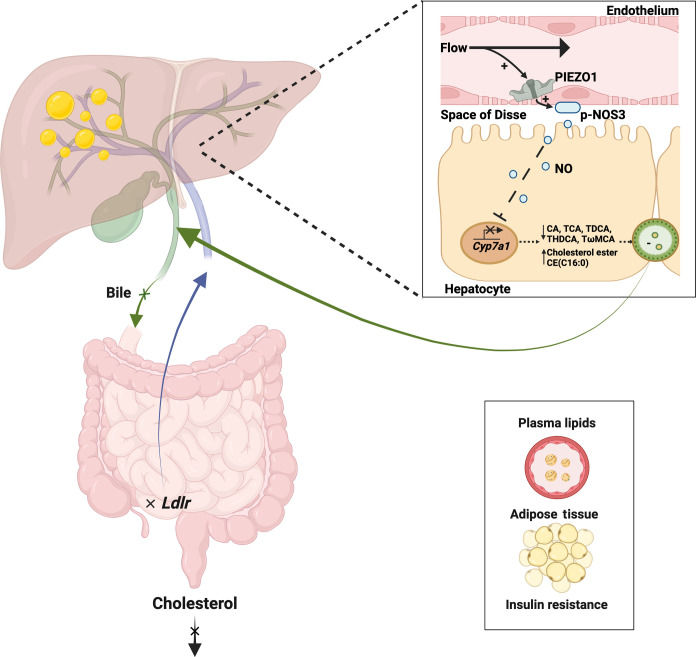
Parenchymal cell lipid regulation by endothelial force sensing. Diagram outlining the hypothesis from the study, which is that endothelial force sensing modulates parenchymal cell lipid regulation. The principle is exemplified by the hepatobiliary system. Blood flows in microvasculature of the liver, generating shear stress at the endothelium of the vascular wall. The shear stress activates PIEZO1 channels, which are force sensors expressed in the endothelial cells. Activation of the channels reduces the expression of *Cyp7a1*, a hepatocyte-restricted mechanism of the parenchyma. Signaling from PIEZO1 to *Cyp7a1* depends on the diffusible messenger, nitric oxide (NO). With reduced expression of *Cyp7a1*, there is reduced capability for catalysis of cholesterol to bile acids, which in turn reduces bile acids produced by the hepatocytes, reduces bile flow in the gallbladder, and increases hepatic cholesterol ester CE:(C16). Similar effects in the small intestine are suggested. *Ldlr* expression in the distal part of the small intestine and fecal cholesterol output are reduced. In addition, plasma lipids are increased along with adipose tissue accumulation and insulin resistance. p-NOS3 eNOS, S1176-phosphorylated endothelial nitric oxide synthase; NO, nitric oxide; *Cyp7a1*, gene encoding Cytochrome P450 family 7 subfamily A member 1; *Ldlr*, gene encoding low-density lipoprotein receptor; CA, cholic acid; TCA, taurocholic acid; TDCA, taurodeoxycholic acid; THDCA, taurohyodeoxycholic acid; TwMCA, tauro-omega-muricholic acid; CE(16,0), cholesteryl palmitate. Created with BioRender.com.

Results from independent murine genetic strategies and portal perfusion experiments in this study suggest that blood flow stimulates mechanically activated PIEZO1 channels in hepatic endothelium to negatively regulate *Cyp7a1* in neighboring hepatocytes via a diffusible messenger, thereby reducing bile acids and increasing cholesterol (Fig. 5). Such a concept of endothelial force sensing that controls the lipid regulatory function of parenchymal cells was expanded to the small intestine where *Ldlr* was regulated and lipid excretion controlled. Efficient exchange of gases and nutrients between blood and tissues requires close proximity of endothelial and parenchymal cells. We suggest that this proximity confers an additional opportunity for blood flow to regulate parenchymal cell function via the sensing of shear stress at the endothelium and its coupling to a diffusible messenger (Fig. 5). In this way, blood flow of cardiovascular activity interconnects with lipid control centers. The idea adds to an expanding picture of the regulation of metabolism by mechanical forces (*56*).

It is likely that endothelial PIEZO1 links to hepatocyte *Cyp7a1* via nitric oxide because PIEZO1 powerfully stimulated phosphorylation of NOS3 in hepatic endothelial cells, and substrate-based inhibition of nitric oxide synthesis blocked PIEZO1’s coupling to *Cyp7a1* (Fig. 5). We combine this information with prior knowledge that nitric oxide negatively regulates *Cyp7a1* through a nitrosylation pathway (*48*, *49*). A further test of the hypothesis could be to try to detect local elevation of nitric oxide between endothelial cells and hepatocytes during portal perfusion. It would be challenging to achieve, however. Nitric oxide is highly unstable, and the techniques for measuring it in such situations are not well developed or definitive. Although we show regulation of NOS3 in hepatic endothelial cells, our data do not exclude roles of other NOS isoforms.

NOS3 is also called endothelial NOS (eNOS), but its expression is not endothelial restricted. It is also expressed in hepatocytes (*50*, *51*, *57*–*59*). Consistent with hepatocyte localization, we observed abundant NOS3 and phosphorylated NOS3 in total liver lysate that was unaffected by endothelial PIEZO1 deletion. These NOS3 signals most likely arose from hepatocytes, the dominant cell type of the liver. *Cyp7a1* would be constitutively inhibited if hepatocyte NOS3 were to be constitutively active, thus potentially making it unavailable. NOS3 is, however, susceptible to multiple phosphorylation events that activate or inhibit it (*60*), so there could be other regulation of hepatocyte NOS3 that leaves *Cyp7a1* available and open to regulation by endothelial NOS3. Further experiments are required to test such a hypothesis. Along similar lines, PIEZO1 is also expressed in multiple cell types (*11*), for example, regulating bile duct activity (*61*).

The effects we observed on whole-body lipids were complex, possibly because altering *Cyp7a1* and *Ldlr* expression has broad-ranging implications and these genes were not the only ones regulated. Moreover, tissues beyond the liver and small intestine may contribute to the systemic phenotype, particularly because of the adipose tissue effect. We focused on *Cyp7a1* and *Ldlr* because of their known important roles and their regulation independent of dietary fat. Complexity is also likely because of homeostatic mechanisms that come into play when expressions of *Cyp7a1* and *Ldlr* change, potentially influencing, for example, the abundance of specific bile acids such as the taurine-conjugated secondary bile acids. Diet-dependent effects were also observed (e.g., Fig. 3C), suggesting actions of endothelial PIEZO1 beyond *Cyp7a1* and *Ldlr*. The regulation of *Slc10a1* only in the HFD condition (table S1) could confer diet-dependent regulation of bile acids (*62*). There was also diet-dependent regulation of *Fgf15* (table S6), which encodes fibroblast growth factor 15 (FGF15), a modulator of *Cyp7a1* and other lipid and carbohydrate regulatory mechanisms (*63*). A potential dietary-dependent effect on the microbiota would require validation in a larger-scale study (fig. S6C). The data suggest superimposition of diet-dependent regulation.

Despite the complexity, endothelial PIEZO1–deleted mice had less cholesterol, less hepatic triglycerides, less plasma triglycerides, and less fatty liver, especially in the HFD condition. All of these effects are potentially beneficial in societies in which the diet is rich in fat. Moreover, the genetic information from human populations suggests importance of PIEZO1 in lipid homeostasis of people. Therefore, further understanding of PIEZO1 mechanisms may point to unique ways of lowering triglycerides and cholesterol, thus protecting against cardiovascular disease (*4*, *5*). Complete genetic ablation of PIEZO1 associates with disease in people (i.e., generalized lymphatic dysplasia), but partial disruption of PIEZO1 appears to be without major adverse effect (*64*). Therefore, partial inhibition of PIEZO1, for example, via pharmacological methods, may be safe. If this direction is to be pursued for therapeutics, we would need progress with PIEZO1 antagonists.

The gene encoding PIEZO1 is highly polymorphic (*65*), suggesting extensive population diversity of its expression and function. About a third of people of recent African ancestry carry a *PIEZO1* variant that confers gain of function (*66*). We studied mice engineered to carry a gain-of-function mutation (PIEZO1^M-R/M-R^) that was originally identified in people (*67*). These mice had increased liver triglycerides. Therefore, people who carry a gain-of-function mutation may have increased risk of dyslipidemia and benefit most should PIEZO1 inhibitors become available as a therapeutic strategy.

Shear stress might not be the only mechanical stimulus that is relevant to this mechanism because PIEZO1 channels are promiscuous sensors of mechanical forces (*11*) that also arise, for example, from matrix stiffness (*68*). There is therefore likely to be increased force on the mechanism proposed as, for example, blood pressure increases with hypertension and tissue stiffness increases with fibrosis and aging. Therefore, PIEZO1 channels and the endothelial-parenchymal units may become mechanically hyperactive through nongenetic as well as genetic mechanisms.

Our findings suggest mechanisms that may contribute to the relationship between physical activity and lipid homeostasis. Whole-body blood flow is redistributed in common types of exercise, increasing in skeletal muscle and decreasing in the liver, small intestine, and some other organs (*8*). Portal flow is reduced in physical activity (*45*). Our data suggest that such a decrease in portal flow will lead to increased expression of *Cyp7a1* and thus increased bile and decreased cholesterol. A mechanism of this type could confer health benefits in people with high plasma cholesterol. Studying the mechanisms is likely to be challenging, however. PIEZO1 is widely expressed in endothelial cells of multiple organs, and organ-specific methods for manipulating it are not yet available. Previous work showed that endothelial PIEZO1 deficiency leads to microvascular rarefaction in skeletal but not cardiac muscle, reducing whole-body physical exercise performance (*35*). Here, we specifically addressed hepatic endothelial function by portal vein cannulation experiments, but manipulation of this type precludes whole-body physical exercise studies in which mice freely run on a wheel or perform other types of exercise.

In conclusion, the data suggest an endothelial force sensing mechanism that regulates lipid homeostasis through signaling to parenchymal cells of lipid control centers such as epithelial cells of the liver and small intestine (Fig. 5). A basis is suggested for understanding how cardiovascular activity interacts with lipid homeostasis. Ideas are suggested for developing therapies for diseases such as cardiovascular diseases that arise from or are exacerbated by hyperlipidemia.

## METHODS

### PIEZO1^∆EC^ mice

All animal use was authorized by the University of Leeds Animal Ethics Committee and The Home Office, UK. Animals were maintained in GM500 individually ventilated cages (Animal Care Systems) at 21°C, 50 to 70% humidity, light/dark cycle 12/12 hours on CD or 60% fat (HFD) (catalog no. F3282) ad libitum, and bedding of Pure-o'Cel (Special Diet Services, Datesand Ltd., Manchester, UK). Genotypes were determined using real-time PCR with specific probes designed for each gene (Transnetyx, Cordova, TN). C57BL/6 J mice with *Piezo1* gene flanked with LoxP sites (*Piezo1*-floxed) were described previously (*17*). To generate tamoxifen (TAM, Sigma-Aldrich)–inducible disruption of *Piezo1* gene in endothelium, *Piezo1*-floxed mice were crossed with mice expressing cre recombinase under the endothelial-specific *Cadh5* promoter [Tg(*Cdh5*-cre/ERT2)]1Rha and inbred to obtain *Piezo1*flox/flox/*Cdh5*-cre mice (*17*). TAM was dissolved in corn oil (Sigma-Aldrich) at 20 mg ml^−1^. Mice were injected intraperitoneally with TAM (75 mg kg^−1^) for five consecutive days, and studies were performed up to 10 weeks later. *Piezo1*flox/flox/*Cdh5*-cre mice that received TAM injections are referred to as PIEZO1^ΔEC^ mice. *Piezo1*flox/flox littermates (lacking *Cdh5*-cre) that received TAM injections were the controls (Control mice).

### PIEZO1^M-R/M-R^ mice

PIEZO1^M-R/M-R^ mice were generated using CRISPR-Cas9 methodology as described elsewhere (*47*). The guide RNA (gRNA) was designed to direct Cas9-mediated cleavage of *Piezo1* 6 bp upstream of the target methionine codon with no off-target sites of less than three mismatches predicted elsewhere in the genome (binding sequence 5′ GGGCGCUCAUGGUGAACAG 3′). A 120-nt single-stranded homology-directed repair (ssHDR) template was designed to incorporate the methionine-to-arginine missense mutation, in addition to silent mutations introducing a Mlu 1 restriction enzyme recognition site to facilitate genotyping (5′ccacccgtcccctgagcctgaggggctccatgctgagcgtgcttccatccccagccaTTAttTacGCGTagcgcccagcagccatccattgtgccattcacaccccaggcctacgaggag 3′; capital letters denote the mutated bases) (Sigma-Aldrich). The gRNA, delivered as an alt-R CRISPR RNA (crRNA) combined with Trans-activated CRISPR RNA (tracrRNA) (Integrated DNA Technologies, Illinois, USA), Cas9 protein (New England Biolabs), and polyacrylamide gel electrophoresis (PAGE)–purified ssHDR template (Integrated DNA Technologies, Illinois, USA) were microinjected into C57BL/6 mouse zygotes and implanted in females (University of Manchester Transgenic Unit). Successful gene editing of pups was identified by Mlu 1 digestion of PCR amplicons 3 (F: 5′ TCTGGTTCCCTCTGCTCTTC 3′, R: 5′ TGCCTTCGTGCCGTA CTG 3′) and confirmed by DNA sequencing following subcloning into pCRTM-Blunt (Invitrogen). Further genotypes were determined by real-time PCR with specific probes designed for each gene (Transnetyx, Cordova, TN). Eight-week-old male mice were used for experiments unless otherwise stated. Only male mice were used for experiments.

### C57Bl6/J mice

Male C57Bl6/J (Charles River Laboratories, UK) mice were purchased at approximately 7 weeks of age. Mice were acclimated to a 12:12-hour light:dark cycle for 2 weeks and were maintained in GM500 individually ventilated cages (Animal Care Systems) at 21°C, 50 to 70% humidity, and light/dark cycle 12/12 hours on CD.

### Collection of blood

Systemic blood was collected in EDTA-coated tubes from the vena cava and centrifuged (4000*g*, 4°C, 10 min). Plasma was aliquoted and snap frozen in liquid nitrogen. Organs were dissected, their weight was recorded, and they were either directly snap frozen in liquid nitrogen or fixed in 4% formalin for 48 hours. Frozen samples were stored at −80°C until analysis.

### RNA extraction and real-time qPCR analysis

Total RNA was prepared from tissues using TRIzol reagent (Sigma-Aldrich). To extract RNA, chloroform was added to the samples. After centrifugation, the aqueous phase was collected, and isopropanol was added to precipitate the RNA. The RNA pellet was washed and suspended in ribonuclease (RNase)–free water. Reverse transcription of mRNA was performed using Superscript III Reverse Transcriptase (Invitrogen) in the presence of random primers (Promega) and RNase OUT (Invitrogen). Briefly, 1 μg of total RNA from each sample was used with 2 μl of random hexamer (20 μM) and 0.5 μl of RNase OUT. After 8 min of incubation at 70°C to inactivate deoxyribonuclease, 100 U of Moloney Murine Leukemia Virus (M-MLV) transcriptase (Invitrogen) was added, and the mixture was incubated at 75°C for 7 min, 10 min at room temperature (RT), and then at 37°C for 1 hour. The RT reaction was ended by heating the mixture at 95°C for 5 min. It was then chilled and stored at −80°C until use. Real-time qPCR analysis was performed using SYBR Green I Master (Roche) on LightCycler 480 Real-Time PCR System. PCRs were run in a 384-well format with 10 μl of reaction mixture containing SYBR Green I Master (Bio-Rad) (5 μl), cDNA from the RT reaction (1 μl), and gene specific primers (1.25 μM, 4 μl). Reactions were run with a standard two-step cycling program, 95°C for 10 s and 60°C for 40 s, for 40 cycles. Each analysis included both housekeeping gene and gene of interest, primer pairs as multiplexed samples. mRNA abundance was calculated relative to the average of the housekeeping gene rps19 and further normalized to the relative expression level of the respective controls (8 to 10 mice per group). The reactions were analyzed with the ΔΔCT analysis method. PCR primers are specified in table S7.

### RNA sequencing and data processing

The quality of RNA samples was assessed using NanoDrop, agarose gel electrophoresis, and Agilent 2100. RNA sequencing was performed by Novogene Co., Ltd., Cambridge using Illumina Novaseq platform with 150-bp paired ends. Raw data were filtered to remove low-quality reads (Qscore ≤5) and adapters. Each sample generated more than 60 million clean reads. Raw reads in FASTQ format were provided by the sequencing facility. The initial quality control of the reads was performed using FastQC v0.118 (*69*). The reads were trimmed to remove low-quality reads, using TrimGalore v0.6.2 (*70*) with a minimum quality score of 20 and at least length of 20 bp. Reads were aligned to the mouse reference genome (GRCm38 *Mus Musculus*) using STAR v2.7 (*71*) aligner. To improve the quality of the data, the alignments only retained uniquely mapped reads. Postalignment quality control was performed. featureCounts v1.6 (*72*) was run with default parameters to quantify the read counts of each gene and used for differential expression analysis. The raw data are deposited in ArrayExpress: E-MTAB-9996.

### Differential expression analysis

Differential gene expression analysis was performed using the R package DESeq2 v1.26 (*73*). Low-expression genes were removed from the analysis. Genes with a sum greater than 10 read counts across all samples were retained in the analysis. *DESeq2* normalizes the raw read counts based on the median of ratios as described previously (*74*). Differentially expressed genes (DEGs) were identified with adjusted *P* value below threshold 0.10 using the Benjamini-Hochberg correction procedure. Volcano plot and heatmap of DEGs were constructed using the ggplot2 package (*75*) and pheatmap function (*76*) implemented in R computing environment (4.3.0.), respectively.

### In situ liver perfusion

Eight- to 12-week-old mice were then anesthetized by intraperitoneal injection of ketamine hydrochloride (100 mg kg^−1^) and xylazine hydrochloride (15 mg kg^−1^). The portal vein was cannulated for inflow and the inferior vena cava for outflow. The liver was perfused for 5 min with phosphate-buffered saline (PBS) to flush out blood and prevent clotting of the hepatic vessels. Liver was then perfused with RPMI 1640 (Gibco 22400089) supplemented with 10% heat-inactivated fetal bovine serum (Sigma-Aldrich, F9665-500ML) and 1% penicillin/streptomycin (Gibco) in a closed circuit for 2 hours with a peristaltic pump (Watson Marlow, 505Di). Flow was applied changing the flow rate of the peristaltic pump: 0.47 or 1.41 ml min^−1^.

### Liver endothelial cell isolation

Liver of 12- to 14-week-old male mice was used, and tissue was mechanically separated using forceps, further cut in smaller pieces and incubated at 37°C for 50 min, in a MACSMix Tube Rotator to provide continuous agitation, along with 0.1% Collagenase II (17101-015, Gibco, Waltham, MA) and Dispase Solution (17105-041, Gibco). Following enzymatic digestion samples were passed through 100- and 40-μm cell strainers to remove any undigested tissue. The suspension was incubated for 15 min with dead-cell removal paramagnetic beads (130-090-101, Miltenyi Biotec GmbH, Bergisch Gladbach, Germany) and then passed through an LS column (130-042-401, Miltenyi Biotec). The cell suspension was incubated with CD146 magnetic beads (130-092-007, Miltenyi Biotec 130-092-007) at 4°C for 15 min under continuous agitation and passed through an MS column (130-042-201, Miltenyi Biotec). The CD146-positive cells, retained in the MS column, were plunge out with PEB [1× PBS, bovine serum albumin (5 g liter^−1^), and 2 mM EDTA] and centrifuged at 1000 rpm for 5 min. Cells were seeded on poly-d-lysine–coated six-well plates and cultured in EGM2 media (C-22211, PromoCell Gmbh) supplemented with EGM2 supplement kit (C-39211, PromoCell Gmbh) until reaching 90% confluency. Cells were then starved in Standard Bath Solution (SBS) for 1.5 hours before treatment with 2 μM Yoda1 or control vehicle (Veh., dimethyl sulfoxide) for 1 min. Cell pellet were lysed with NP40 lysis buffer [10 mM tris (pH 7.4), 150 mM NaCl, 0.5 mM EDTA (pH 8.0), 0.5% Nonidet P40, containing phosphatase inhibitor (P0044, Sigma-Aldrich), and protease inhibitor (P8340, Sigma-Aldrich)].

### Gallbladder cannulation

Mice were then anesthetized by intraperitoneal injection of ketamine hydrochloride (100 mg kg^−1^) and xylazine hydrochloride (15 mg kg^−1^). The common bile duct was ligated close to the duodenum, and then the gallbladder was punctured and cannulated with a polyethylene-10 catheter. Bile was collected for 30 min, after a stabilization time of 30 min. During bile collection, body temperature was stabilized using a temperature mattress. Bile flow (μl min^−1^ 100 g of body weight^−1^) was determined gravimetrically assuming a density of 1 g ml^−1^ for bile. Aliquots were snap frozen for later analysis.

### Immunoblotting

Livers were harvested and snap frozen in liquid nitrogen. Total liver lysate was prepared as 5% homogenate in SET buffer [250 mM sucrose, 1 mM EDTA, and 10 mM tris-HCl (pH 7.5)] containing both phosphatase (P0044, Sigma-Aldrich) and protease (P8340, Sigma-Aldrich) inhibitors for later analysis by immunoblotting. Protein concentration was determined using the bicinchoninic acid (BCA) assay, and 20 μg of total protein was heated at 95°C for 5 min in SDS-PAGE sample buffer and then loaded on a precast 4 to 20% polyacrylamide gradient gel (4561094, Bio-Rad) and subjected to electrophoresis. Proteins were transferred onto a polyvinylidene difluoride membrane using semidry transfer for 30 min. Membranes were blocked with 5% milk in tris-buffered saline with Tween 0.05% for 1 hour at RT. The membranes were exposed to primary antibody overnight at 4°C (phospho-eNOS S1176: BD 612392; total eNOS/NOS3: BD 610296; anti-mouse: Jackson ImmunoResearch 715-035-150; β-actin: Santa Cruz Biotechnology sc4778), then rinsed and incubated with appropriate horseradish peroxidase–labeled secondary antibody for 1 hour at RT. The detection was performed by using SuperSignal West Femto (34096, Thermo Fisher Scientific), and immunoblots were visualized with a G-Box Chemi-XT4 (SynGene, Cambridge, UK). β-Actin was used as the reference protein.

### In vivo injection of l-NMMA

Twenty-week-old mice were injected intraperitoneally with 10 mM l-NMMA (N^G^-methyl-l-arginine acetate salt, Tocris Bioscience, 0771) to reach a final systemic concentration of 10 μM. Liver and distal small intestine tissues were harvested 4 hours later. mRNA was isolated as described above.

### Liquid chromatography–mass spectrometry lipid quantification

A solution of 10 μM palmitoyl-l-carnitine-(*N*-methyl-d3) (Sigma-Aldrich), 10 μM palmitic acid-d31 (Sigma-Aldrich), and 10 μM deoxycholic acid-d6 (Sigma-Aldrich) in liquid chromatography–mass spectrometry (LC-MS)–grade methanol was prepared as internal standard spiking solution (ISSS). Samples (from gall bladder cannulation) were reconstituted in 100 μl of LC-MS-grade water and 100 μl of ISSS, vortex mixed, and centrifugated for 3 min at 16,000xrcf before being transferred to LC vials. Chromatography was performed using an Acquity UPLC system (Waters) equipped with a CORTECS T3 2.7-μm (2.1 mm by 30 mm) column, which was kept at 60°C. The Acquity UPLC system was coupled to a Xevo TQ-XS mass spectrometer (Waters). The binary solvent system used was solvent A comprising LC-MS–grade water, 0.2 mM ammonium formate, and 0.01% formic acid and solvent B comprising analytical-grade acetonitrile/isopropanol 1:1, 0.2 mM ammonium formate, and 0.01% formic acid. For all analyses, a 10-μl injection was used, and the mobile phase was set at a flow rate of 1.3 ml min^−1^. For bile acid analysis, the column mobile phase was held at 20% solvent B for 0.1 min, followed by an increase from 20 to 55% solvent B over 0.7 min. The mobile phase was increased to 98% solvent B and held for 0.9 min. The mobile phase was then returned to 20% solvent B held for 0.1 min to re-equilibrate the column. Analyses were performed using multiple reaction monitoring (*77*).

### Microbiome composition analysis

Fresh fecal pellets from mice were collected before culling mice and snap frozen until analysis. Frozen fecal pellets were transferred to Transnetyx microbiome sample collection tubes to be later submitted to the Transnetyx automatic whole-genome sequencing platform. Sequencing was done using shallow shotgun sequencing methodology (Illumina NovaSeq, at a depth of 2 million 2 × 150-bp read pairs), enabling species and strain taxonomic resolution. Data visualization of taxonomy was performed at the One Codex microbiome analysis platform (One codex, San Francisco, CA). Alpha diversity was analyzed using Shannon, Chao1 and Simpson indexes. Beta diversity analysis was done using Bray Curtis. Both alpha and beta diversity data were performed in Jupyter Notebooks environment. Taxa classification was performed in One Codex on relative abundances species.

### UK Biobank, FinnGen, Cardiovascular Disease Knowledge Portal, and NHGRI-EBI GWAS catalog

The chromosome 16 genomics data from UK Biobank was obtained under the identifier 42651. The data were filtered to excluded participants with more than 10% missing data, exclude variants with Hardy-Weinberg equilibrium less than 1 × 10^−6^ and exclude variants with minor allele frequency less than 5% for common variants or less than 1% for less frequent variants, using PLINK version 2.0 software. The related hepatobiliary disease terms were obtained using the ID-20002 self-reported noncancer illnesses code. Logistic regression analyses were performed using PLINK2 and adjusted for effects of sex, age, and the first 10 principal components. In FinnGen, we accessed the publicly available data using FinnGenn r7 (https://www.finngen.fi/en/access_results). Then, we screened for the PIEZO1 mutations linked to hepatobiliary diseases. This release contains 3095 disease endpoints. The CVD knowledge portal data are publicly available https://cvd.hugeamp.org/ as is the EBI catalog www.ebi.ac.uk/gwas/.

### Measurement of neutral lipids

Lipids from 2 mg of liver or 100 mg of feces were extracted according to previously described methods (*78*) in dichloromethane/methanol/water (2.5/2.5/2.1, v/v/v), in the presence of internal standards: 4 g of stigmasterol, 4 g of cholesteryl heptadecanoate, and 8 g of glyceryl trinonadecanoate. The dichloromethane phase was evaporated to dryness and dissolved in 30 μl of ethyl acetate. The lipid extract (1 μl) was analyzed by gas-liquid chromatography on a FOCUS Thermo Electron system using a Zebron-1 Phenomenex fused silica capillary columns (5 m by 0.32 mm inside diameter, 0.50-μm film thickness) as previously described (*79*). Oven temperature was programmed from 200° to 350°C at a rate of 5°C/min, and the carrier gas was hydrogen (0.5 bar). The injector and the detector were at 315° and 345°C, respectively.

### Measurement of plasma lipids

Total cholesterol and triglycerides (TGs) and HDL cholesterol concentrations were measured with commercial kits (CHOD-PAP for cholesterol no. 87656, GPO-PAP for TGs no. 87319 and Cholesterol HDL-PTA for HDL cholesterol no. 86516; BIOLABO at ABLIANCE, Compiègne, France). Nonesterified fatty acid concentrations were measured using a kit from WAKO chemicals.

### Measurement of plasma bile acids and salts

Plasma (100 μl) was diluted in 400 μl of acetonitrile (ACN) in the presence of an internal standard (23NorDCA, 20 ng). The mixture was agitated for 30 min at RT and centrifuged for 5 min at 10,000 rpm. The supernatant was concentrated and reconstituted in 1 ml of 15 mM ammonium acetate (pH 5.3). The lipid extracts were preconcentrated on HLB Solid-Phase Extraction (Waters SAS, Saint-Quentin-en-Yvelines). After conditioning of the plate (1 ml of water, 2 ml of methanol), samples were loaded, and plates were washed with 2 ml of water, and last, bile acids were eluted with 2 ml of methanol. After concentration, final extracts were solubilized in 20 μl of methanol and kept at −20°C until the analysis by LC-MS in high resolution. They were run on an Ultimate 3000 (Thermo Fisher Scientific–Life Technologies SAS, Saint-Aubin, France) equipped with a ZORBAX SB-C18 column (2.1 mm by 100 mm, 1.7 μm, Agilent, Santa Clara, USA) kept at 45°C*.* Mobile phases were composed of (A) 15 mM ammonium acetate (pH 5.3) (acetic acid), (B) acetonitrile, and (C) methanol, in gradient mode at 0.5 ml min^−1^. The gradient was as follows: 20% B/0%C at 0 min until 2.5 min 16% B/20%C at 5 min; 1% B/95%C at 20 min; 1% B/95%C at 23 min; 20% B/1%C at 23.5 min until 27.5 min. The injection volume was 5 μl. High-resolution mass spectrometry detection was done on an Exactive (Thermo Fisher Scientific, Life Technologies SAS, Saint-Aubin, France) in an electrospray ionization–negative mode and an acquisition in full-scan mode from 200 to 600 mass/charge ratio. Parameter of the source were as follows: sheath gas: 206,843 Pa, auxiliary gas flow: 68,947.6 Pa, sweep as flow rate: 0 Pa, spray voltage: 2500 V, capillary temperature: 200°C, capillary voltage: −37.5°C, tube lens voltage: −165 V, heater temperature: 200°C, skimmer voltage: −46 V, maximum injection time: 250 ms. Calibration curves containing all the pure standards in methanol were run at different concentration (24, 6, 1.5, 0.375, 0.093, 0.023, and 0.0058 ng ml^−1^). Quantification was carried out using the ion chromatogram obtained for each compound using 5-ppm windows with Trace Finder Quantitative Soft Ware (Thermo Fisher Scientific–Life Technologies SAS, Saint-Aubin, France). The methodology was adapted from a previously described approach (*80*).

### Tissue staining

Liver tissues were frozen in O.C.T. compound (Tissue-Tek), and 10-μm frozen liver sections were air dried for 30 min and fixed in 4% formaldehyde. Oil Red O staining was performed using standard protocols. Tissue slides were scanned using an Axio Scan.Z1 on the Bioimaging platform at the University of Leeds. Oil Red O staining was quantified using ImageJ software.

### Glucose and insulin tolerance tests

For glucose tolerance tests, mice were fasted overnight before intraperitoneal injection with 1 mg kg^−1^ glucose. For insulin tolerance tests, mice were fasted for 2 hours before intraperitoneal injection with recombinant human insulin (0.75 U kg^−1^; Actrapid; Novo Nordisk). Blood was collected from the tail vein at time 0 and after 30, 60, 90, and 120 min for determination of glucose levels. Blood glucose was measured with a portable glucometer.

### Indirect calorimetry

Combined Laboratory Animal Monitoring System (CLAMS) (Columbus Instruments) was used to monitor oxygen consumption, carbon dioxide production, food intake, and locomotory activity using Oxymax software (version 5.37.05, Columbus Instruments). The CLAMS was calibrated before each experiment. Animals were subjected to a 2-day acclimation period in a training cage to habituate to the environment of the metabolic cages. Animals were maintained in normal bedding at 22°C throughout the monitoring period. Ten-minute interval measurements for each animal were obtained for oxygen and carbon dioxide with ad libitum access to food and water (or water plus metabolites) on a controlled 12-hour light/dark cycle. Cages contained one mass sensor to monitor food intake. Data were analyzed using CaIR (version 1.1).

### Statistical analysis

Genotypes of mice were always blinded to the experimenter, and mice were studied in random order determined by the genotype of litters. Datasets comparing control and PIEZO1^ΔEC^ mice were analyzed for statistical significance by independent Student’s *t* test without assuming equal variance. Outliers were removed in the validation analysis using the robust regression and outlier removal test method with *Q* = 1% in GraphPad Prism 9.0 software. Statistical significance was considered to exist at probability *P*: *P < 0.05, **P < 0.01, or ***P < 0.001. When data comparisons are shown without an asterisk, there were no significantly differences. The number of independent experiments is indicated by *n*. Descriptive statistics are shown as means ± SD unless indicated otherwise. All underlying individual data points are shown superimposed on mean data. Origin Pro software was used for data analysis and presentation unless otherwise indicated.

### Ethics statement

All work with mice occurred under the authority of the University of Leeds Animal Welfare and Ethical Review Committee and UK Home Office Project Licences P606320FB and PP8169223.

## Supplementary Materials

### The PDF file includes:

Figs. S1 to S6 Tables S1 to S8 Uncropped Western blots Legend for data file S1
