# Supplementary material for: Endothelial force sensing signals to parenchymal cells to regulate bile and plasma lipids
Source: Sci Adv. 2024 Sep 27;10(39):eadq3075. doi: 10.1126/sciadv.adq3075 (PMC11430402; doi:10.1126/sciadv.adq3075)
Supplement: Supplementary file 1 — Figs. S1 to S6 Tables S1 to S8 Uncropped Western blots Legend for data file S1 [file sciadv.adq3075_sm.pdf]

Science Advances
AAAS

Supplementary Materials for

Endothelial force sensing signals to parenchymal cells to regulate bile and plasma lipids

Laeticia Lichtenstein *et al.*

Corresponding author: Laeticia Lichtenstein, l.lichtenstein@leeds.ac.uk; David J. Beech, d.j.beech@leeds.ac.uk

Sci. Adv. **10**, eadq3075 (2024)
DOI: 10.1126/sciadv.adq3075

The PDF file includes:

Figs. S1 to S6
Tables S1 to S8
Uncropped Western blots
Legend for data file S1

Other Supplementary Material for this manuscript includes the following:

Data file S1

SUPPLEMENTAL MATERIALS

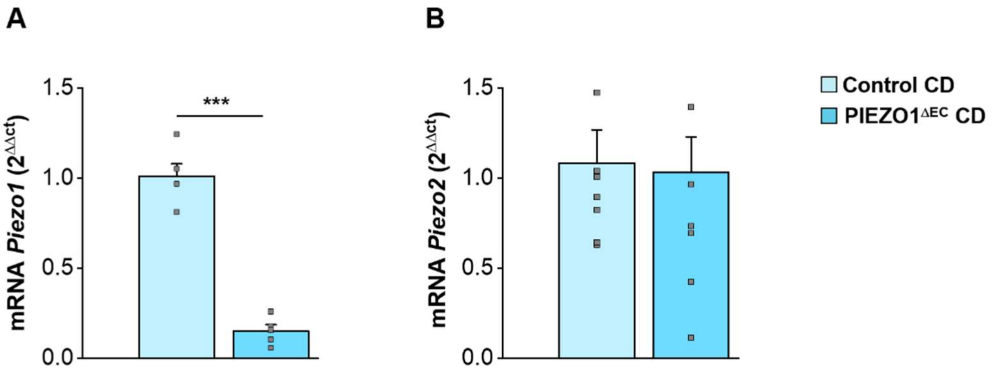

Figure S1 Validation of *Piezo1* disruption. mRNA abundance for (**A**) *Piezo1* (n = 6) and (**B**) *Piezo2* (n = 8) in primary liver endothelial cells of control and PIEZO1$^{\Delta EC}$ mice, after 20 weeks, normalised to *rps19* mRNA abundance. Summary data are mean ± s.d.. Bar charts show the underlying individual data superimposed as open symbols. Some individual points are overlapping. Unpaired T-Test. Statistically significant difference: ****P*<0.001.

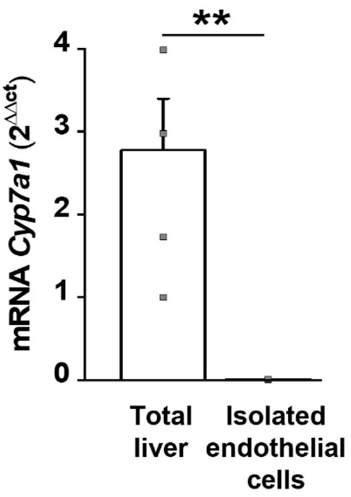

Figure S2 Lack of expression of *Cyp7a1* in isolated liver endothelial cells. mRNA abundance for *Cyp7a1* in total liver (n = 5) and primary liver endothelial cells (n = 5) of wild-type mice, normalised to *rps19* mRNA abundance. Summary data are mean ± s.d.. Bar charts show the underlying individual data superimposed as open symbols. Some individual points are overlapping. Unpaired T-Test. Statistically significant difference: $^{**}P<0.01$.

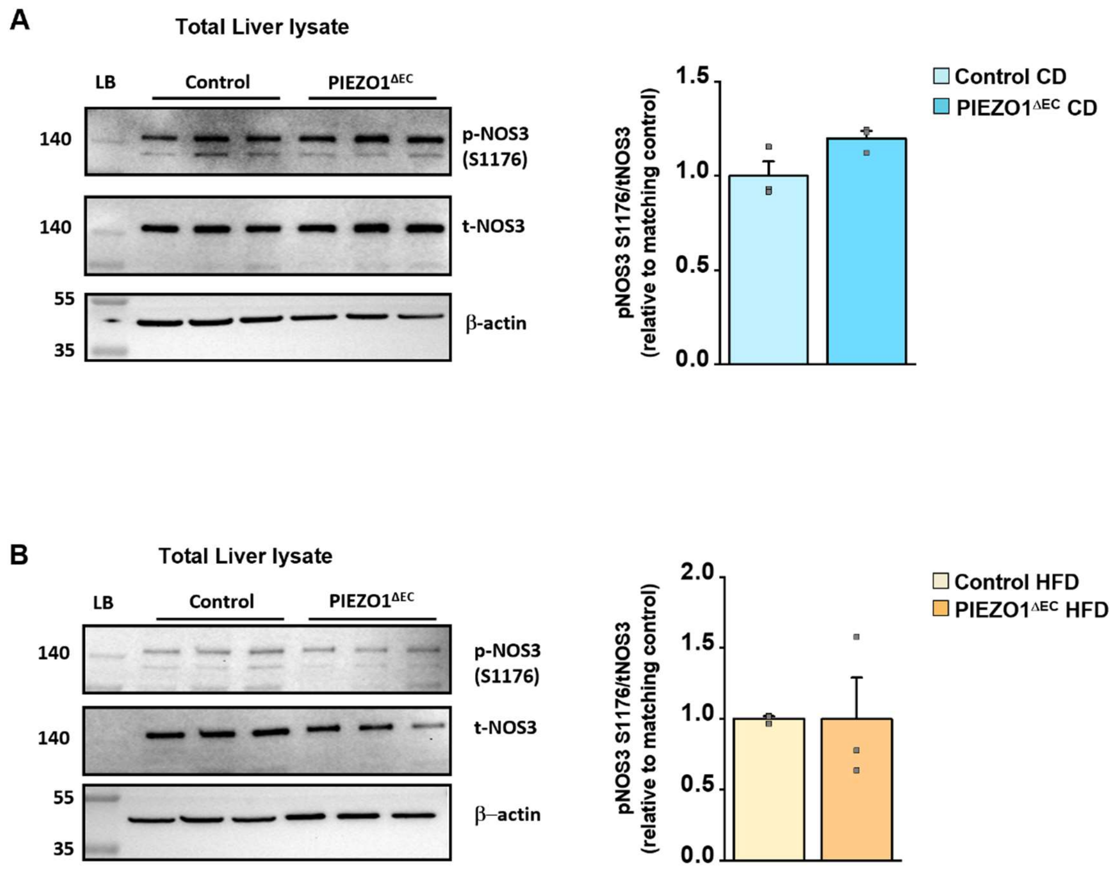

Figure S3 Immunoblot quantification of NOS3 on total liver lysate. Measurements were made from 20 week-old mice fed chow diet (CD) and then (**A**) CD (data in blue) or (**B**) high fat diet (HFD, data in orange) for 8 weeks. Light coloured data indicate data from control mice and darker coloured data indicate data from PIEZO1$^{\Delta EC}$ mice. On the left, example Western blot for total liver lysate proteins from control and PIEZO1$^{\Delta EC}$ mice. Blots were probed for NOS3 phosphorylation at S1176 (p-NOS3), total NOS3 (t-NOS3) and the loading control protein, β-actin. On the right, quantification for experiments of the type exemplified, expressed as phospho-NOS3 intensity relative to total NOS3 intensity and normalised to matching Vehicle condition (n = 3 each group). LB: Loading buffer. Summary data are mean ± s.d.. Charts show the underlying individual data superimposed as open symbols. Some individual points are overlapping. Unpaired T-Test.

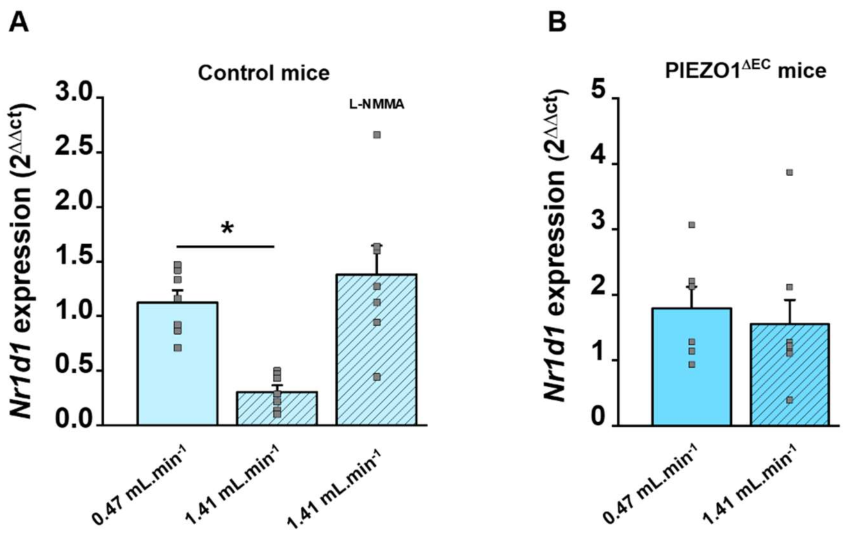

Figure S4 *Nr1d1* expression in perfused liver. Bar charts showing (**A**) liver *Nr1d1* Q-PCR data from liver perfusion experiments at two perfusion rates for control mice normalised to *rps19* mRNA abundance (light blue, 0.47 $mL.min^{-1}$, n = 7 mice; light blue hatched, 1.41 $mL.min^{-1}$, n = 6 mice; light blue hatched, L-NMMA at 1.41 $mL.min^{-1}$, n = 7 mice) and (**B**) $PIEZO1^{\Delta EC}$ mice at 0.47 $mL.min^{-1}$ (n = 6) and at 1.41 $mL.min^{-1}$ (n = 8). Summary data are mean ± s.d.. Bar charts show the underlying individual data superimposed as open symbols. Unpaired T-Test. Statistically significant difference: **P*<0.05.

A

GLM

Effect	Full Day Mass	Full Day Group	Full Day Interaction	Light Mass	Light Group	Light Interaction	Dark Mass	Dark Group	Dark Interaction
Hourly Food Consumed (kcal)	0.4680	0.1934		0.8878	0.0786		0.3763	0.3159	
Total Food Consumed (kcal)	0.6681	0.2189		0.7774	0.2437		0.5911	0.2021	
Oxygen Consumption (ml/hr)	0.5736	0.6103		0.3677	0.4954		0.8595	0.7713	
Carbon Dioxide Production (ml/hr)	0.6554	0.7334		0.2215	0.5376		0.7553	0.9694	
Energy Expenditure (kcal/hour)	0.5895	0.5915		0.3374	0.4510		0.9400	0.7912	
Energy Balance (kcal/hour)	0.4371	0.1923		0.6344	0.0647		0.3986	0.3256	

ANOVA

Effect	Full Day Group	Light Group	Dark Group
Respiratory Exchange Ratio	0.6774	0.7310	0.7131
Locomotor Activity (beam breaks)	0.0692	0.1099	0.0528
Ambulatory Activity (beam breaks)	0.3137	0.3490	0.3076

B

GLM

Effect	Full Day Mass	Full Day Group	Full Day Interaction	Light Mass	Light Group	Light Interaction	Dark Mass	Dark Group	Dark Interaction
Hourly Food Consumed (kcal)	<0.001 ***	0.2109		0.0073 **	0.8151		0.0012 **	0.0493 *	
Total Food Consumed (kcal)	0.0052 **	0.7348		0.0024 **	0.6438		0.0079 **	0.8048	
Oxygen Consumption (ml/hr)	0.2144	0.6244		0.5630	0.6365		0.1392	0.5926	
Carbon Dioxide Production (ml/hr)	0.7621	0.8312		0.9249	0.8939		0.7108	0.7292	
Energy Expenditure (kcal/hour)	0.2920	0.6281		0.6599	0.6464		0.2002	0.5885	
Energy Balance (kcal/hour)	<0.001 ***	0.2902		<0.001 ***	0.4914		<0.001 ***	0.0665	

ANOVA

Effect	Full Day Group	Light Group	Dark Group
Respiratory Exchange Ratio	0.3477	0.2721	0.4706
Locomotor Activity (beam breaks)	0.7072	0.4519	0.9440
Ambulatory Activity (beam breaks)	0.5181	0.3836	0.6847

Mass effect: subject.mass.x

Signif. codes: <0.001 ***,<0.01 **,<0.05 *

Figure S5 CLAMS data in support of Figure 4(H-O). Generalized linear model (GLM) and ANOVA (analysis of variance) table generated by CalR (web-based tool for analysis of indirect calorimetry experiments) from CD group (**A**) and HFD group (**B**) from Oxymax Comprehensive Laboratory Animal Monitoring System (CLAMS). Time Plots tab for data visualization of individual metabolic parameters for the selected period, during full day, light and dark photoperiods. Statistically significant difference: *P<0.05; **P<0.01; ***P<0.001.

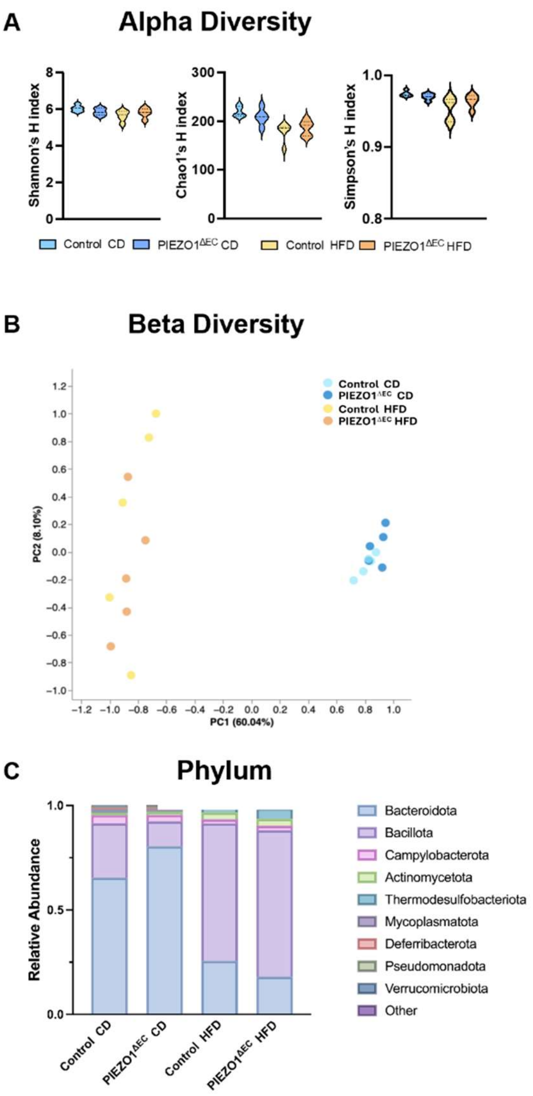

Figure S6 Microbiome analysis. (**A**) Alpha diversity indices (Shannon, Chao1 and Simpson). (**B**) Beta diversity Bray Curtis. (**C**) Stacked bar plot showing distribution of microbiome at phylum level between the four groups. One way ANOVA shows only dietary effect, unpaired student T-Test in CD groups shows significant differences in Bacteroidota and Bacillota relative abundance, suggesting a possible effect of PIEZO1 profile in CD (n=5 per group).

Table S1 Expression of genes regulating bile acid metabolism. Gene expression of hepatic genes involved in bile acid homeostasis in liver of control and PIEZO1$^{\Delta EC}$ mice fed a chow diet and High fat diet. mRNA expression was normalised to *rps19* mRNA abundance. Gene were grouped according to their contribution to bile acid synthesis, bile acid export, bile acid reabsorption, bile acid conjugation and bile acid signalling. *Akr1d1, Cyp27a1, Abcc2, Abcc3, Nr0b2, Fgfr4* (CD: control n= 12; PIEZO1$^{\Delta EC}$ n= 10; HFD: control n= 12; PIEZO1$^{\Delta EC}$ n= 12); *Cyp8b1, Abcb11, Slc10a1, Slco1a1, Slc27a5, Baat, Nr1h4* (CD n=14; HFD n=15); *Cyp2c70* (CD control n= 14; PIEZO1$^{\Delta EC}$ n= 13; HFD n=12). Data are expressed in 2 $^{\Delta\Delta CT}$. Unpaired T-Test. Statistically significant difference: *P<0.05.

Gene	Control CD	PIEZO1$^{\Delta EC}$ CD	*P* value	Control HFD	PIEZO1$^{\Delta EC}$ HFD	*P* value
Bile acid synthesis						
Akr1d1	1.23 ± 0.21	1.25 ± 0.24	0.94	1.25 ± 0.20	0.89 ± 0.09	0.12
Cyp8b1	1.15 ± 0.14	1.02 ± 0.14	0.38	1.06 ± 0.18	0.84 ± 0.08	0.32
Cyp2c70	4.09 ± 1.41	3.82 ± 1.29	0.91	1.00 ± 0.44	0.57 ± 0.30	0.43
Cyp27a1	1.27 ± 0.31	1.18 ± 0.23	0.82	1.23 ± 0.18	1.26 ± 0.15	0.88
Bile acid Export						
Abcb11	1.05 ± 0.09	0.89 ± 0.10	0.13	1.17 ± 0.16	1.11 ± 0.9	0.76
Abcc2	1.07 ± 0.10	0.99 ± 0.11	0.62	0.96 ± 0.13	1.09 ± 0.07	0.37
Bile acid reabsorption						
Abcc3	1.08 ± 0.13	1.07 ± 0.13	0.96	1.84 ± 0.41	1.95 ± 0.29	0.82
Slc10a1	1.09 ± 0.14	1.24 ± 0.18	0.66	1.06 ± 0.11	0.75 ± 0.05	0.02 *
Slco1a1	1.18 ± 0.18	1.21 ± 0.17	0.76	1.16 ± 0.17	1.32 ± 0.11	0.44
Bile acid conjugation						
Slc27a5	1.13 ± 0.16	1.15 ± 0.13	0.88	1.08 ± 0.13	1.04 ± 0.09	0.80
Baat	1.39 ± 0.38	1.81 ± 0.42	0.63	1.96 ± 0.49	2.10 ± 0.55	0.85
Bile Acid Signalling						
Nr1h4	1.18 ± 0.18	1.58 ± 0.30	0.40	1.38 ± 0.25	1.21 ± 0.18	0.59
Nr0b2	1.51 ± 0.46	1.13 ± 0.20	0.49	1.24 ± 0.19	1.43 ± 0.29	0.60
Fgfr4	1.16 ± 0.22	1.04 ± 0.13	0.66	1.22 ± 0.18	1.38 ± 0.20	0.57

Table S2 Bile acid species in PIEZO1$^{\Delta EC}$ mice. Bile acid secretions in Chow fed control and PIEZO1$^{\Delta EC}$ mice, in µM/min/100g of body weight (bw). CA: Cholic acid (cholate); TCA: Taurocholic acid (taurocholate); GCA: Glycocholic acid; CDCA: Chenodeoxycholic Acid; TCDCA: TauroChenodeoxycholic Acid; GCDCA: Glyco Chenodeoxycholic Acid; GUDCA: Glycolursodeoxycholic acid; βMCA: beta-muricholic acid; TαMCA: Tauro-alpha-muricholic acid; TβMCA: Tauro-beta–muricholic acid; DCA: deoxycholic acid; TDCA: Taurodeoxycholic acid (taurodeoxycholate); GDCA: Glycodeoxycholic acid; TLCA: Taurolithocholic acid; GLCA: glycolythocholic acid; THDCA: Taurohyodeoxycholic acid; ωMCA: omega-muricholic acid; TωMCA: Tauro-omega-muricholic acid (ω-tauromuricholate) (n = 8 each group). Summary data are mean ± s.d.. Unpaired T-Test. Statistically significant differences: *P<0.05; **P<0.01.

Bile acids (mM/min/100g bw)	Control CD	PIEZO1$^{\Delta EC}$ CD	*P* value
CA	0.908 ± 0.26	1.51 ± 0.46	0.002**
TCA	6.66 ± 0.58	16.91 ± 2.38	0.01*
GCA	1175.39 ± 990.60	2928.45 ± 3997.82	0.25
CDCA	22.87 ± 23.40	35.06 ± 41.75	0.48
TCDCA	0.46 ± 0.23	0.56 ± 0.25	0.46
GCDCA	0.63 ± 0.83	1.83 ± 2.64	0.24
GUDCA	0.57 ± 0.36	1.19 ± 1.73	0.34
βMCA	0.02 ± 0.01	0.08 ± 0.07	0.04*
TαMCA	205.95 ± 105.79	260.58 ± 116.10	0.34
TβMCA	0.089 ± 0.47	1.29 ± 1.15	0.37
DCA	12.87 ± 16.22	24.42 ± 32.46	0.38
TDCA	0.19 ± 0.06	0.71 ± 0.63	0.04*
GDCA	236.61 ± 228.63	797.47 ± 1173.25	0.21
TLCA	42.24 ± 47.05	89.55 ± 123.01	0.33
GLCA	4.41 ± 4.49	12.12 ± 16.06	0.21
THDCA	0.54 ± 0.22	1.28 ± 0.93	0.04*
ωMCA	0.58 ± 0.37	1.22 ± 1.75	0.33
TωMCA	15324.63 ± 6311.73	27288.99 ± 10060.61	0.01*

Table S3 Bile acid species in PIEZO1$^{MR/MR}$ mice. Bile acid secretions in Chow fed PIEZO1$^{WT/WT}$ (Control) and PIEZO1$^{MR/MR}$ mice, in µM/min/100g of body weight (bw). CA: Cholic acid; TCA: Taurocholic acid; GCA: Glycocholic acid; CDCA: Chenodeoxycholic Acid; TCDCA: TauroChenodeoxycholic Acid; GCDCA: Glyco Chenodeoxycholic Acid; GUDCA: Glycoursodeoxycholic acid; βMCA: beta-muricholic acid; TαMCA: Tauro-alpha-muricholic acid; TβMCA: Tauro-beta-muricholic acid; DCA: deoxycholic acid; TDCA: Taurodeoxycholic acid; GDCA: Glycodeoxycholic acid; TLCA: Taurolithocholic acid; GLCA: Glycolythocholic acid; THDCA: Taurohyodeoxycholic acid; ωMCA: omega-muricholic acid; TωMCA: Tauro-omega-muricholic acid (PIEZO1$^{WT/WT}$ n = 8 PIEZO1$^{MR/MR}$ n = 7). Summary data are mean ± s.d.. Unpaired T-Test. Statistically significant differences: *P<0.05; **P<0.01.

Bile acids (mM/min/100g bw)	PIEZO1$^{WT/WT}$ CD	PIEZO$^{MR/MR}$ CD	*P* value
CA	2.15 ± 0.52	1.35 ± 0.34	0.004**
TCA	10.65 ± 7.25	11.51 ± 8.80	0.84
GCA	5882.91 ± 4233.42	1661.74 ± 1288.26	0.03*
CDCA	117.84 ± 260.87	9.17 ± 8.46	0.29
TCDCA	0.98 ± 0.55	0.45 ± 0.20	0.03*
GCDCA	0.84 ± 0.70	0.69 ± 0.62	0.68
GUDCA	2.96 ± 1.96	0.70 ± 0.64	0.01*
βMCA	0.03 ± 0.03	0.04 ± 0.07	0.69
TαMCA	483.71 ± 342.57	191.51 ± 133.15	0.05
TβMCA	1.13 ± 0.51	1.21 ± 0.66	0.82
DCA	11.85 ± 244.51	6.58 ± 6.72	0.28
TDCA	0.38 ± 0.56	0.18 ± 0.20	0.37
GDCA	465.32 ± 419.32	283.72 ± 258.57	0.34
TLCA	70.83 ± 60.66	36.22 ± 32.18	0.20
GLCA	7.60 ± 6.67	3.98 ± 3.71	0.23
THDCA	1.39 ± 0.54	0.69 ± 0.18	0.01*
ωMCA	3.02 ± 2.00	0.76 ± 0.66	0.01*
TωMCA	20972.93 ± 1344.83	18166.81 ± 8075.12	0.60

Table S4 Additional gene expression data for L-NMMA-treated mice. Q-PCR mRNA expression data for 13 genes in liver of wildtype mice injected with 10 μM L-NMMA or vehicle control as described for Figure 2c. For each gene, L-NMMA data are shown as the fold-change relative its respective vehicle control data, presented as mean ± s.d. with underlying individual data for each mouse superimposed as open symbols. There were no statistically significant differences between L-NMMA and vehicle groups (n = 5 per group). Unpaired T-Test.

Gene name	**Vehicle**	**L-NMMA**	*P* value
Cd36	1.00 ± 0.26	1.06 ± 0.50	0.81
Dgat2	1.00 ± 0.17	0.94 ± 0.12	0.53
Mttp	1.00 ± 0.12	0.92 ± 0.16	0.38
Fasn	1.00 ± 0.24	1.09 ± 0.77	0.80
Ppara	1.00 ± 0.17	0.79 ± 0.22	0.13
Ppard	1.00 ± 0.20	0.91 ± 0.11	0.37
Hmgcr	1.00 ± 0.43	1.14 ± 0.25	0.54
Acat2	1.00 ± 0.12	0.86 ± 0.14	0.13
Abcg8	1.00 ± 0.15	0.93 ± 0.16	0.48
Abcb11	1.00 ± 0.31	1.01 ± 0.22	0.97
Slc10a1	1.00 ± 0.13	1.10 ± 0.46	0.64
Vldlr	1.00 ± 0.31	0.91 ± 0.62	0.77

Table S5 Bile acid species in plasma of PIEZO1$^{\Delta EC}$ mice. Plasma bile acid quantification, expressed in percentage of total bile acid detected in PIEZO1$^{\Delta EC}$ mice vs control, chow diet and HFD fed in % of total plasma bile acid. CA: Cholic acid; TCA: Taurocholic acid; βMCA: beta-muricholic acid; TαMCA: Tauro-alpha-muricholic acid; TβMCA: Tauro-beta-muricholic acid; alloLCA: allolythocholic acid; DCA: deoxycholic acid; TDCA: Taurodeoxycholic acid; ωMCA: omega-muricholic acid, (n = 6 each group). Summary data are mean ± s.d.. Unpaired T-Test. Statistically significant difference: *P<0.05.

	Chow Diet (CD)			High Fat Diet (HFD)		
Bile acid (%)	Control	PIEZO1$^{\Delta EC}$	*P* value	Control	PIEZO1$^{\Delta EC}$	*P* value
CA	25.51 ± 14.98	10.83 ± 9.76	0.152	9.41 ± 5.26	8.23 ± 2.03	0.690
TCA	26.54 ± 12.65	57.47 ± 22.87	0.025 *	30.56 ± 27.92	17.99 ± 20.74	0.462
βMCA	15.05 ± 8.47	11.28 ± 7.37	0.474	10.12 ± 10.00	20.77 ± 16.24	0.201
TαMCA	11.42 ± 16.02	11.77 ± 9.89	0.981	6.42 ± 0.14	8.69 ± 5.15	0.582
TβMCA	8.26 ± 7.85	15.57 ± 13.57	0.404	4.42 ± 6.19	18.56 ± 13.47	0.046 *
alloLCA	9.08 ± 14.91	10.88 ± 11.31	0.88	5.16 ± 6.49	3.48 ± 6.17	0.65
DCA	13.11 ± 10.07	6.75 ± 6.45	0.31	5.44 ± 9.36	5.35 ± 2.62	0.98
TDCA	1.30 ± 0.81	1.94 ± 1.75	0.59	Trace amounts	Trace amounts	ND
ωMCA	9.36 ± 9.51	4.45 ± 2.99	0.36	48.31 ± 22.15	27.97 ± 15.75	0.097

Table S6 Expression of bile acid and cholesterol metabolism-related genes in small intestine of PIEZO1$^{\Delta EC}$ mice. Gene expression of intestinal genes involved in bile acid homeostasis of control and PIEZO1$^{\Delta EC}$ mice fed a chow diet and High fat diet. mRNA expression was normalised to *rps19* mRNA abundance. Gene were grouped according to their contribution to bile acid signalling, bile acid absorption, bile acid reabsorption, bile acid export, cholesterol transport, cholesterol signalling and fatty acid metabolism. (CD: control n= 12; PIEZO1$^{\Delta EC}$ n= 10; HFD: control n= 11; PIEZO1$^{\Delta EC}$ n= 12). Data are expressed in 2 $^{\Delta\Delta CT}$. Summary data are mean ± s.d.. Unpaired T-Test. Statistically significant difference: *P<0.05; **P<0.01.

Gene name	Control CD	PIEZO1$^{\Delta EC}$ CD	*P* value	Control HFD	PIEZO1$^{\Delta EC}$ HFD	*P* value
Bile acid signalling						
Fgf15	1.18 ± 0.21	2.48 ± 0.20	0.03*	25.6 ± 3.64	13.2 ± 0.08	0.02*
Bile Acid absorption						
Asbt	1.01 ± 0.08	1.03 ± 0.12	0.91	0.96 ± 0.16	1.25 ± 0.28	0.37
Bile Acid Reabsorption						
Ibapt	1.01 ± 0.08	1.02 ± 0.12	0.91	2.37 ± 0.39	3.07 ± 0.67	0.36
Ostb	1.01 ± 0.07	0.84 ± 0.07	0.08	2.67 ± 0.51	3.03 ± 0.27	0.58
Abcc3	1.05 ± 0.09	0.96 ± 0.09	0.52	2.04 ± 0.17	1.85 ± 0.22	0.52
Bile acid Export						
Abcc2	0.99 ± 0.08	0.91 ± 0.10	0.49	5.85 ± 0.43	5.41 ± 0.37	046
Cholesterol transport						
Npc1l1	1.04 ± 0.09	0.69 ± 0.04	0.003**	1.08 ± 0.13	1.07 ± 0.13	0.95
Scarb1	1.06 ± 0.12	1.03 ± 0.09	0.81	1.80 ± 0.86	1.95 ± 0.44	0.81
Abcg5	1.07 ± 0.08	1.05 ± 0.12	0.88	1.46 ± 0.24	1.34 ± 0.24	0.72
Abcg8	1.12 ± 0.15	1.29 ± 0.13	0.40	1.30 ± 0.15	1.06 ± 0.15	0.28
Cholesterol signalling						
Nr1h3	1.01 ± 0.06	0.94 ± 0.05	0.33	2.15 ± 0.19	1.99 ± 0.18	0.55
Fatty acid metabolism						
Cd36	1.06 ± 0.15	0.99 ± 0.10	0.68	0.82 ± 0.17	1.09 ± 0.31	0.45
Apob100	1.32 ± 0.24	1.13 ± 0.14	0.48	2.14 ± 0.51	2.66 ± 0.85	0.60
Mttp	1.03 ± 0.08	0.77 ± 0.06	0.09*	1.28 ± 0.13	1.38 ± 0.23	0.72
Acat2	0.98 ± 0.13	0.97 ± 0.06	0.96	1.04 ± 0.13	0.97 ± 0.13	0.73

Table S7 *PIEZO1* variants associated with hepatobiliary and dyslipidaemia phenotypes. P-values for *PIEZO1* single nucleotide polymorphism (SNP) associations with hepatobiliary phenotypes in people from FinnGen, Cardiovascular Disease Knowledge Portal catalog. rsID: reference SNP cluster ID. All SNPs are in intronic sequence.

rsID	*P*-value	Phenotype	Database	Variant type
rs6500500	$1.80x10^{-04}$	Toxic liver disease	FinnGen	Intron
rs561783880	$7.60x10^{-04}$	Non-alcoholic fatty liver disease	FinnGen	Intron
rs9933309	$5.14x10^{-07}$	LDL cholesterol	CVDKP	Intron
rs2608604	$6.39x10^{-07}$	LDL cholesterol	CVDKP	Intron
rs2932690	$3.34x10^{-06}$	LDL cholesterol	CVDKP	Intron
rs2911463	$3.92x10^{-06}$	LDL cholesterol	CVDKP	Intron
rs9932423	$7.24x10^{-06}$	LDL cholesterol	CVDKP	Intron
rs2002833	$1.44x10^{-05}$	LDL cholesterol	CVDKP	Intron
rs8052231	$1.81x10^{-05}$	LDL cholesterol	CVDKP	Intron
rs2911460	$1.91x10^{-05}$	LDL cholesterol	CVDKP	Intron
rs8052370	$3.04x10^{-05}$	LDL cholesterol	CVDKP	Intron
rs2926772	$5.98x10^{-05}$	LDL cholesterol	CVDKP	Intron
rs2932690	$6.15x10^{-08}$	Total cholesterol	CVDKP	Intron
rs2608604	$9.36x10^{-08}$	Total cholesterol	CVDKP	Intron
rs8052231	$9.57x10^{-08}$	Total cholesterol	CVDKP	Intron
rs9932423	$2.02x10^{-07}$	Total cholesterol	CVDKP	Intron
rs8052370	$4.42x10^{-07}$	Total cholesterol	CVDKP	Intron
rs9933309	$5.17x10^{-07}$	Total cholesterol	CVDKP	Intron
rs2002833	$7.47x10^{-07}$	Total cholesterol	CVDKP	Intron
rs2911460	$1.15x10^{-06}$	Total cholesterol	CVDKP	Intron
rs57953994	$3.17x10^{-06}$	Total cholesterol	CVDKP	Intron
rs475596	$4.27x10^{-06}$	Total cholesterol	CVDKP	Intron
rs10445033	$8.42x10^{-06}$	Total cholesterol	CVDKP	Intron
rs889764	$1.21x10^{-05}$	Total cholesterol	CVDKP	Intron
rs6500505	$1.22x10^{-05}$	Total cholesterol	CVDKP	Intron
rs2911463	$1.38x10^{-05}$	Total cholesterol	CVDKP	Intron
rs3803578	$2.82x10^{-05}$	Total cholesterol	CVDKP	Intron
rs889761	$2.96x10^{-05}$	Total cholesterol	CVDKP	Intron
rs2926772	$3.29x10^{-05}$	Total cholesterol	CVDKP	Intron
rs889765	$3.77x10^{-05}$	Total cholesterol	CVDKP	Intron
rs113974433	$5.46x10^{-05}$	Total cholesterol	CVDKP	Intron
rs71158757	$5.53x10^{-05}$	Total cholesterol	CVDKP	Intron
rs2341001	$7.18x10^{-05}$	Total cholesterol	CVDKP	Intron
rs3848236	$7.61x10^{-05}$	Total cholesterol	CVDKP	Intron

Table S8 Nucleotide sequences of Q-PCR primers used in the study

Gene	Forward	Reverse
Rps29	GTCTGATCCGCAAATACGGG	AGCCTATGTCCTTCGCGTACT
Abcb11	CTGCCAAGGATGCTAATGCA	CGATGGCTACCCTTTGCTTCT
Apob100	AAGCACCTCCGAAAGTACGTG	CTCCAGCTCTACCTTACAGTTGA
Asbt	ACCACTTGCTCCACACTGCTT	CGTTCCTGAGTCAACCCACAT
Abcg5	TGCCCATTCCTTTAAAAATCC	GATGAACTGGACCCCTTGG
Abcg8	GGGGCTGATGCAGATTCA	GTAGCTGATGCCGATGACAA
Acat2	TCTTCTTCGCCTTCCTGCACTG	GAAGTCGAGTTCCACCAATCCC
Cd36	GAGCAACTGGTGGATGGTTT	GCAGAATCAAGGGAGAGCAC
Cyp7a1	CAGGGAGATGCTCTGTGTTCA	AGGCATACATCCCTTCCGTGA
Dgat2	GCGCTACTTCCGAGACTACTT	GGGCCTTATGCCAGGAAACT
Fabp1	ATGAACTTCTCCGGCAAGTACC	CTGACACCCCCTTGATGTCC
Fasn	TCCTGGGAGGAATGTAAACAGC	CACAAATTCATTCACTGCAGCC
Fgf15	ACGTCCTTGATGGCAATCG	GAGGACCAAAACGAACGAAATT
Hmgcr	AGCTTGCCCGAATTGTATGTG	TCTGTTGTGAACCATGTGACTTC
Ldlr	GCATCAGCTTGGACAAGGTGT	GGGAACAGCCACCATTGTTG
Mttp	ATACAAGCTCACGTACTCCACT	TCCACAGTAACACAACGTCCA
Npc1l1	CAACATCTTCATCTTTGTTCTTGAG	GCAATGTGAGCCTCTCG
Nr1h3	AGAGATGTCCTTGTGGCTGGAG	TCCACAACTCCGTTGCAGAATCAG
Pcsk9	GTGGTGATTGGATTGAGGCCATAG	CCCAAATGCATTGAGGGCCTTG
Piezo1	TGAGCCCTTCCCCAACAATAC	CTGCAGGTGGTTCTGGATATAG
Piezo2	AATCAAACCAACATTCCCCTTCA	CAGGTAGACGAGCAAAGGAGA
Ppara	TATTCGGCTGAAGCTGGTGTAC	CTGGCATTTGTTCCGGTTCT
Ppard	GACCAGAACACACGCTTCCT	CCGACATTCCATGTTGAGG
Scarb1	CGCCGACCCTGTGTTGTC	GGATGTCTAGGAACAAGGAATGCT
Slc10a1	ATGACCACCTGCTCCAGCTT	GCCTTTGTAGGGCACCTTGT
Slc27a2	ACAACATTCGTGCCAAGTCTCT	CTCCTCCACAGCTTCTTGTAGATC
Vldlr	TCCTGATTGCGAAGACGGTTCTG	ATGCGGCATGTTCTCATATGGC

Uncropped western blots for Figure 2A

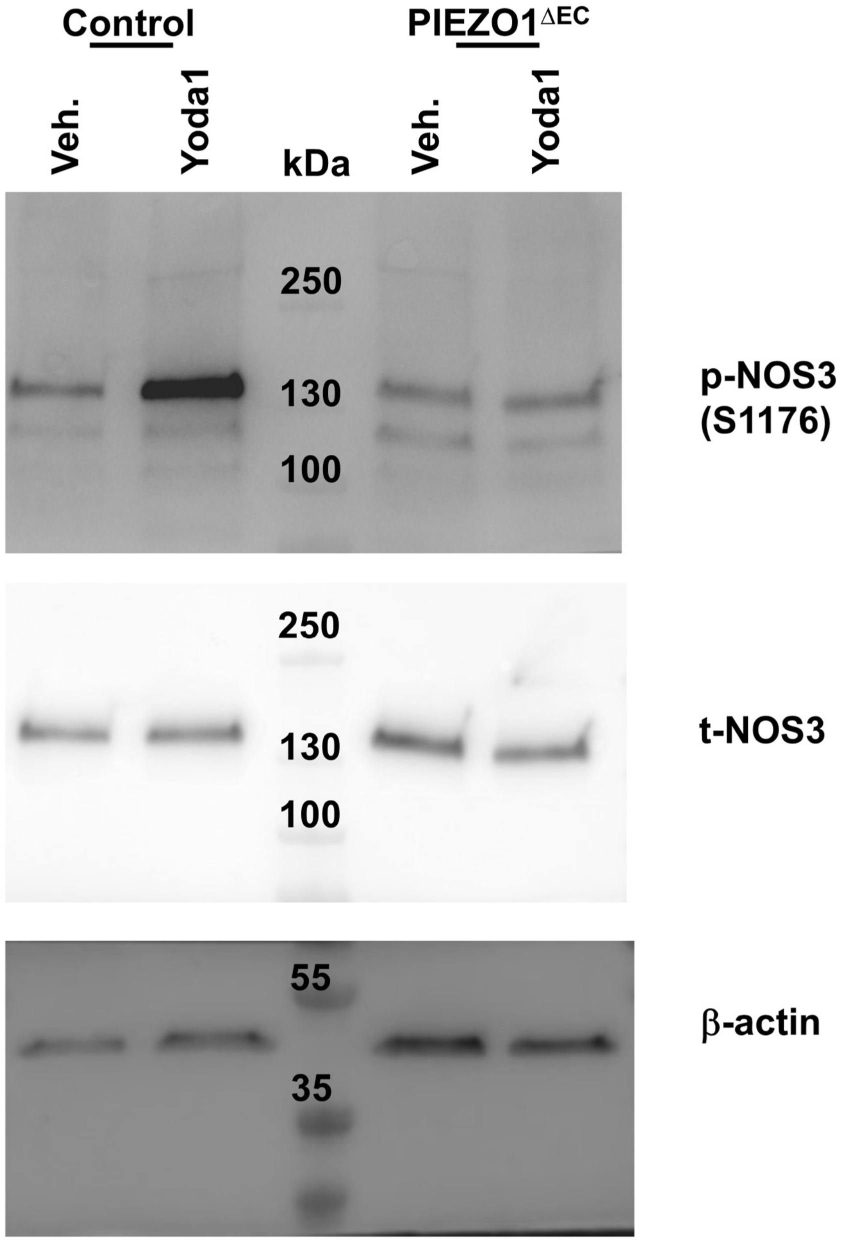

Uncropped western blots for Figure S3

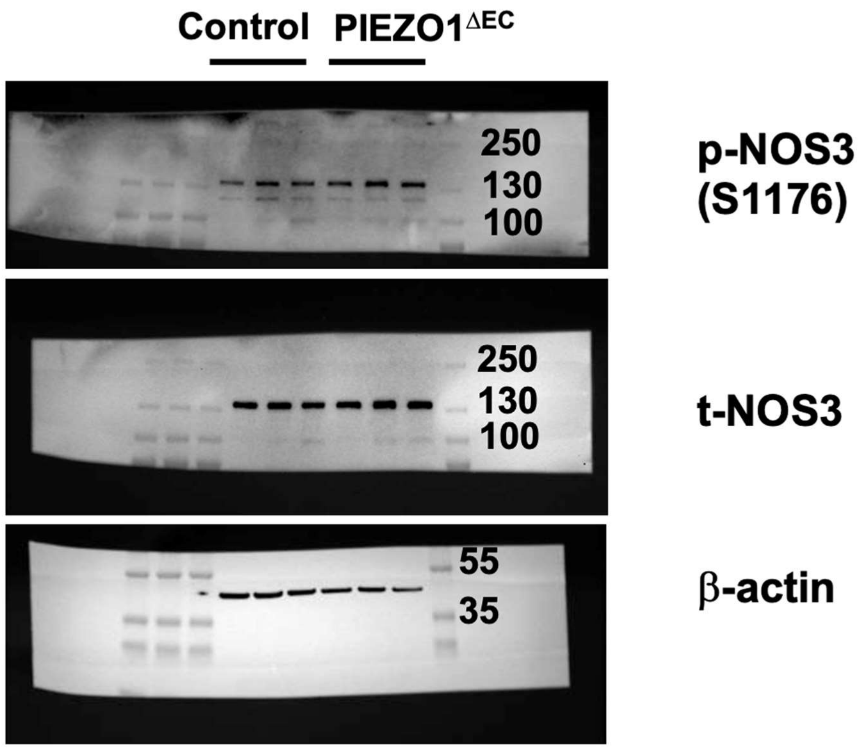

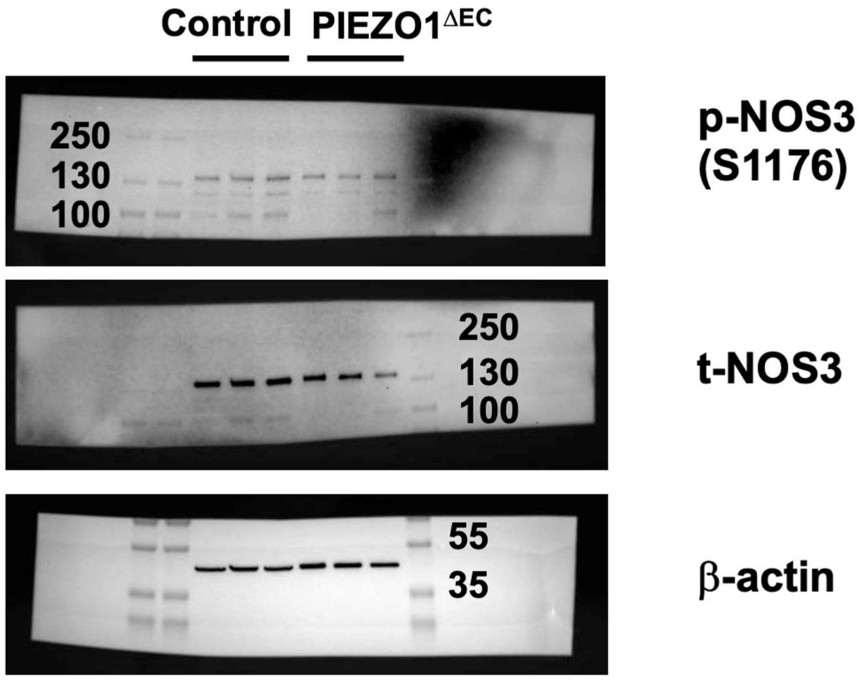

Supplementary data file. Excel file displaying all sources data for the main figures 1A, 1C, 2A-C, 3A-G, 4A-O, supplemental figures S1A, S1B, S2, S3A, S3B, S4A, S4B, S6A, S6C and Tables S1, S2, S3, S4, S5 and S6.
